# Comparative transcriptome analysis between patient and endometrial cancer cell lines to determine common signaling pathways and markers linked to cancer progression

**DOI:** 10.18632/oncotarget.28161

**Published:** 2021-12-21

**Authors:** Madelaine J. Cho-Clark, Gauthaman Sukumar, Newton Medeiros Vidal, Sorana Raiciulescu, Mario G. Oyola, Cara Olsen, Leonardo Mariño-Ramírez, Clifton L. Dalgard, T. John Wu

**Affiliations:** ^1^Department of Gynecologic Surgery & Obstetrics, Uniformed Services University of the Health Sciences, Bethesda, MD 20814, USA; ^2^Collaborative Health Initiative Research Program, Uniformed Services University of the Health Sciences, Bethesda, MD 20814, USA; ^3^National Center for Biotechnology Information, National Library of Medicine, National Institutes of Health, Bethesda, MD 20894, USA; ^4^Preventive Medicine and Biostatistics, Uniformed Services University of the Health Sciences, Bethesda, MD 20814, USA; ^5^National Institute on Minority Health and Health Disparities, National Institutes of Health, Bethesda, MD 20814, USA; ^6^Department of Anatomy, Physiology and Genetics, Uniformed Services University of the Health Sciences, Bethesda, MD 20814, USA

**Keywords:** endometrial cancer, cancer stage, comparative transcriptome analysis, signaling pathways, normalization

## Abstract

The rising incidence and mortality of endometrial cancer (EC) in the United States calls for an improved understanding of the disease's progression. Current methodologies for diagnosis and treatment rely on the use of cell lines as models for tumor biology. However, due to inherent heterogeneity and differential growing environments between cell lines and tumors, these comparative studies have found little parallels in molecular signatures. As a consequence, the development and discovery of preclinical models and reliable drug targets are delayed. In this study, we established transcriptome parallels between cell lines and tumors from The Cancer Genome Atlas (TCGA) with the use of optimized normalization methods. We identified genes and signaling pathways associated with regulating the transformation and progression of EC. Specifically, the LXR/RXR activation, neuroprotective role for THOP1 in Alzheimer’s disease, and glutamate receptor signaling pathways were observed to be mostly downregulated in advanced cancer stage. While some of these highlighted markers and signaling pathways are commonly found in the central nervous system (CNS), our results suggest a novel function of these genes in the periphery. Finally, our study underscores the value of implementing appropriate normalization methods in comparative studies to improve the identification of accurate and reliable markers.

## INTRODUCTION

Endometrial cancer (EC) is a common gynecologic malignancy in the United States with an estimated 66,570 new cases and 12,940 deaths in 2021 [1]. Historically, while EC is presented more commonly amongst older women, it is the only gynecologic cancer with increased incidences at earlier age onset with a concomitant rise in mortality rate [2–5]. Current staging approaches for tumors are important in assessing size, spread, prognosis, and treatment of the disease. Reports that analyzed the Surveillance Epidemiology, and End Results (SEER) database suggest that early tumor staging (stage I and stage II) is correlated to better prognosis and a higher 5-year overall survival rate (OS) in comparison to advanced stages (stage III and stage IV) [1]. The survival rates drop dramatically from 96 to 18–70% respectively [1, 6]. Although the OS is relatively high with early stage detection, the vast majority of late stage EC exhibits a dramatic decline in survival due to lowered responses to radiation, hormone, and non-hormone based treatments [7]. However, staging can be inaccurate and limited in predicting responses to therapy, as some of the early stage lesions display aggressive metastatic behavior, tumor heterogeneity, ambiguous histology, and overlapping molecular characteristics [8–16].

Over the past few decades, cell lines have been frequently used as models to understand cancer biology in tumors [17–20]. With the advent of various platforms and bank centers for next-generation sequencing, large sets of molecular profiles are available for comparison between tumor samples and cell lines [21–23]. Cell lines with maximal molecular similarity to tumors can be useful in identifying targets and signaling mechanisms necessary for drug development [17]. However, a majority of these comparative molecular profiling studies between cell lines and tumors in EC have reported their findings based on integrated genomic characterization (e.g., copy number alternations, polymerase epsilon (POLE) ultramutations, microsatellite instability); whereas analyses in transcriptomics related to stage advancement still remains obscure [24–33]. This is a limitation of targeted genomic approaches due to lack of substantiation at the level of expression and function. Therefore bridging the gap between gene mutations-alterations and transcriptional activity between cancer stages can provide a more comprehensive insight into the processes involved in EC progression.

Here we present a comparative transcriptome analysis between early and advance stage endometrial carcinomas in cell lines and patient tumor samples from the TCGA database. Initially we ascertained whether there are overall transcriptome parallels between cells lines and tumors. Once similarities were established, we identified signaling pathways and potential molecular markers that define changes in progression between early and advanced stage EC. These molecular insights into tumor classification and progression may have a direct effect on providing a more accurate stage classification for EC.

## RESULTS

### Removal of unwanted variation in RNA-seq data

Preliminary findings using scatter plots with linear regression analysis of overall transcriptome for TCGA patients vs cell lines indicates a global shift in favor for higher overall expression for TCGA patients at each stage with R^2^ < 0.46 (Supplementary Table 1; Supplementary Figure 1, left panel). This bias between data sets suggests the existence of unwanted technical effects and the need for a more effective normalization procedure. For this purpose, comparisons between library size and removal of unwanted variation by control genes (RUVg) normalization methods was further evaluated using relative log expression (RLE) and principal component analysis (PCA).

**Figure 1:**
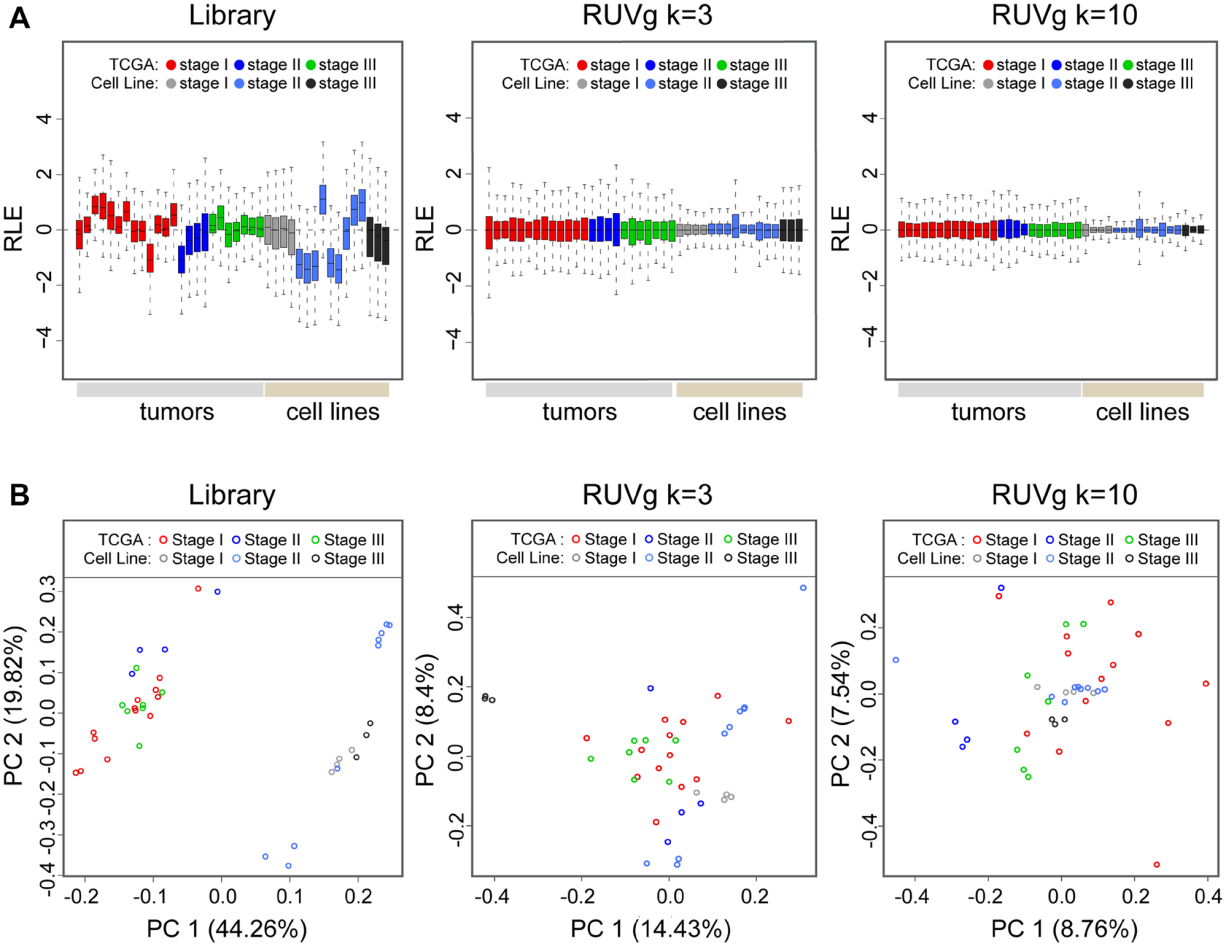
Relative log expression (RLE) and principal component analysis (PCA) plots of overall transcriptome profiles of TCGA tumor samples and endometrial cancer cell lines. (**A**) The RLE boxplot distributions of datasets normalized using RUVg k = 3 or k = 10 resulted in improved log counts centered around zero; demonstrating lowered magnitude in variability and higher resilience toward outliers between tumor samples and cell lines. (**B**) PCA plot axes represents major sources of variation based on genes profiles in the first two dimensions, PC1 and PC2 (centered, log scale)). Scatter plots indicates normalization by RUVg methods leads to better clustering between TCGA tumor samples and cell lines. Normalization with library size, as seen by a distinct separation in scatter plots between TCGA patients and cell lines, suggests similarities in expression are more dependent on sample type.

As seen previously in our findings, normalization of read counts using library size demonstrated to be unsatisfactory. The RLE boxplots displays distributional differences and excessive variability between samples (Figure 1A, left panel). In contrast, the RUVg normalization method resulted in shifting distributions of all read counts across all samples towards 0. Furthermore, an attenuation of expression magnitude suggests improved resilience against outliers (Figure 1A, middle and right panel). This in turn led to a more robust differential expression result downstream (Supplementary Table 2). Due to increase in statistical sensitivity with increasing k value (k = 10), a slight reduction in the number of differentially expressed genes (DEGs), approximately 11–13%, was also observed.

The PCA modeling of overall transcriptome expression offers a global assessment of similarity between samples. The values of first two principal components PC1 (44.26, 14.43, and 8.76%) and PC2 (19.82, 8.40, 7.54%) between library size, RUVg k = 3, and RUVg k = 10 normalization method respectively demonstrated a reduction in variation in expression between samples when using the RUVg method (Figure 1B). Furthermore, when considering normalization by library size, a biological divergence inferred by differential clustering between TCGA patient tumors and cell lines samples was also observed (Figure 1B, left panel). In contrast, the RUVg normalization method eliminated this separation and exhibited scatter plots clustering towards the center indicating greater similarity in overall transcriptome profile between samples (Figure 1B, middle and right panel).

### Differential gene expression analysis

**Figure 2:**
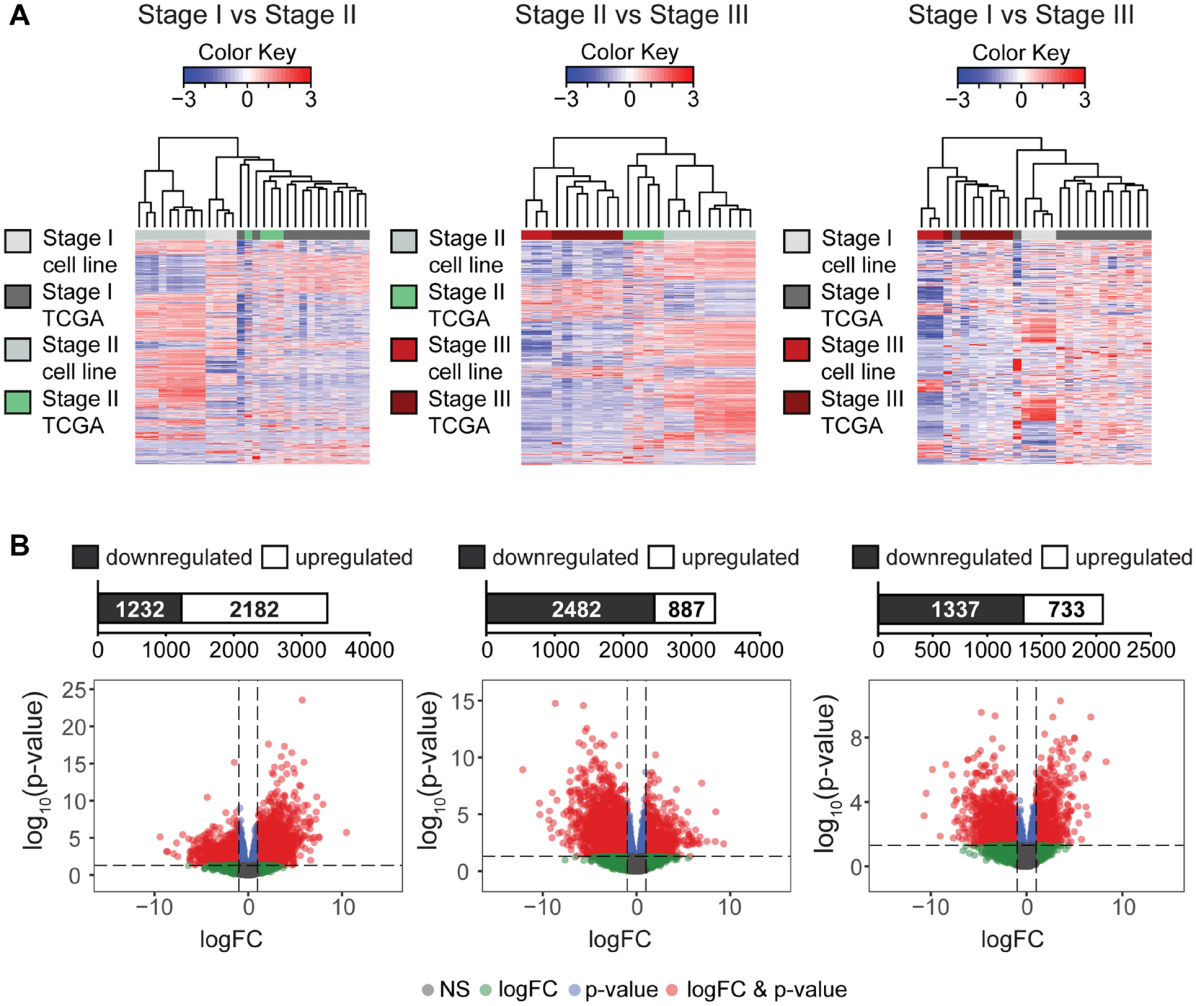
Differential expression analysis of transcriptomes in TCGA tumor samples and endometrial cancer cell lines. (**A**) Hierarchiral clustering analysis of all DEGs between TCGA tumors and cell lines indicates that early stage comparisons show higher degree of clustering between sample types. However, comparisons between early to a later cancer stage demonstrates clustering between stages suggesting clear distinctive expression differences that is stage dependent. All DEGs shown are significant (*p* value <0.05) (top panel). (**B**) Respective volcano plot and bar charts highlighting up- or downregulated DEGs (red dots) suggests downregulation of DEGs with advanced cancer stage (*p* < 0.05; at least |log_2_ fold change (FC)| ≥ 1), non-significant (NS, grey), log_2_ fold change (FC)| ≥ 1 (logFC, green), *p* < 0.05 (*p*-value, blue) (bottom panel).

With the exception of stage I and stage II comparison (Figure 2A, left panel), the hierarchical heat map of the tumor-derived endometrial cancer cell lines and their respective TCGA patient samples clustered together in a stage dependent manner (Figure 2A, middle and right panel). This demonstrates that changes in expression between early and late stage EC in both tumors and cell lines are dependent on staging and not dependent on sample type. The number of DEGs (FDR < 0.05) between each stage comparisons are summarized in Supplementary Table 2.

In order to better understand genes that may be driving stage progression in EC, we identified a set of genes for each stage comparison by merging cell lines and TCGA patients according to stage and using volcano plots that highlights DEGs that are up- or downregulated (*p* < 0.05; at least |log2 fold change (FC)| ≥ 1). For stage I vs. stage II comparison, we observed a differential expression signature of 3,414 genes (2,182 up-regulated and 1,232 down-regulated genes; Figure 2B, left panel). In stage II vs. stage II comparison, we observed 3,369 DEGs (887 up-regulated and 2,482 down-regulated genes; Figure 2B, middle panel). In stage I vs. stage III comparison, we observed 2,070 DEGs (733 up-regulated and 1,337 down-regulated genes; Figure 2B, right panel). The shift from up- to downregulation in global expression from early to late cancer stage suggests a possible divergent mode of action. Gene sets correlated to each stage comparisons and expression directionality are described in Supplementary Table 3.

### Ingenuity pathway analysis (IPA) and gene set enrichment analysis

**Figure 3:**
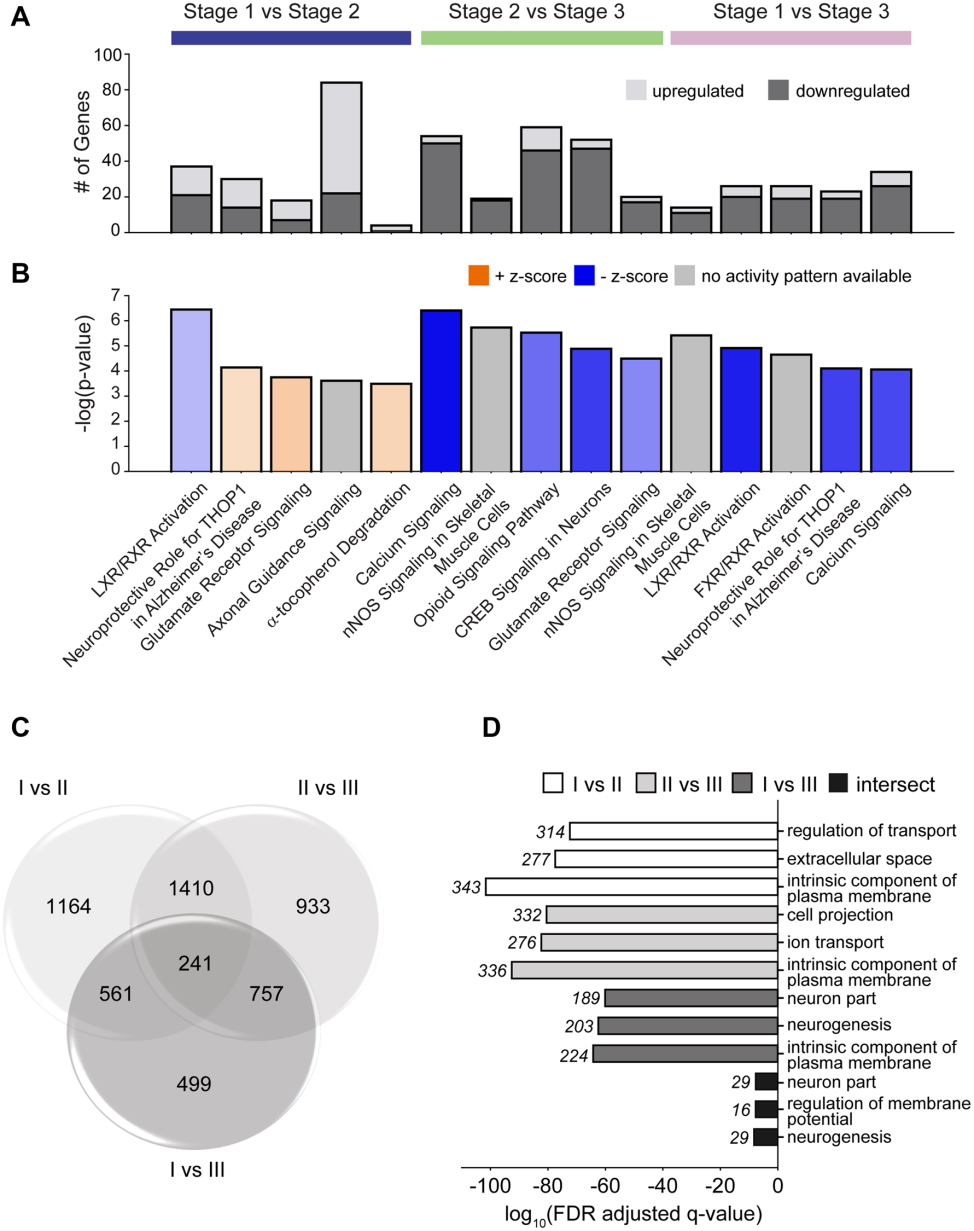
Top signaling pathways and enriched gene sets associated to stage comparisons. (**A**) Top five signaling pathways that are altered between stages. (**B**) Respective z-scores and color intensity that is correlated to expected relationship direction (gene expression from knowledge base) and observed gene expression (**C**) Venn diagram identifying DEGs that are unique or commonly regulated across or between all stages. (**D**) GO analysis of all dysregulated gene sets (FDR <0.05).

In order to determine signaling pathways that are involved in cancer progression, DEGs from each stage comparison in Figure 2B, were used as input for the IPA Core analysis. We identified the top five signaling pathways (*p*-value < 0.05) for each comparison with its respective number of genes that were up- or downregulated (Figure 3A). Out of the fifteen pathways identified, a third appear to be conserved: (1) liver X receptor/retinoid X receptor (LXR/RXR activation), (2) neuroprotective role of THOP1 gene in Alzheimer’s disease, (3) glutamate receptor signaling, (4) nNOS signaling in skeletal muscle cells, and (5) calcium signaling pathways. The majority of these signaling pathways, specifically in the later stage comparisons, appear to be downregulated with a negative z-score indicating a divergent expression direction relationship from the Ingenuity Pathway Knowledge Base (IPKB) (Figure 3B). All other significant pathways are listed in Supplementary Table 4. All genes identified under each signaling pathways are described in Supplementary Table 5.

Further insights into the biological relevance and mechanism of these expression profiles were ascertained using a Venn diagram with subsequent gene set enrichment analysis (GSEA) (FDR <0.05) to identify genes sets within each zone of the Venn diagram. Across all stage comparisons, 241 genes are identified as conserved (intersect), suggesting dynamic expression changes in that set. (Figure 3C; Supplementary Table 6). The identification of the top three significantly enriched gene ontology (GO) gene sets with each stage comparison demonstrates enrichment for regulation of transport, extracellular space, intrinsic component of plasma membrane, cell projection, ion transport, neuron part, neurogenesis, and regulation of membrane potential (Figure 3D). All other gene sets in each of the seven zones are identified in Supplementary Table 6.

## DISCUSSION

In this study, we established transcriptome similarities between the cell lines used in this study and patient tumor samples from TCGA database. This comparison allowed us to identify signaling pathways and gene sets that are dysregulated in both datasets. Most notably, an altered expression pattern in neuronal related signaling pathways and markers was observed between early and advanced histological stages. This expression pattern indicates a novel function of these genes in the periphery with a potential role in regulating the transformation and progression of tumors in EC.

Identifying mutually dysregulated biomarkers and signaling pathways in cell lines and tumors can advantageously provide a more expedient method for studying mechanisms in cancer biology. However, these comparative type studies have proven to be relatively unsuccessful due to the high degree of variability between datasets [18, 20, 23]. Furthermore, inherent variation in tumor collection, processing, and storage between specimens can add an additional layer of variability within the tissue data set [34]. The current standard practice of RNA-seq normalization by library size may be inadequate for complex data sets involving varied samples, platforms, library kits, sequencing depth, and users [35]. Previous efforts have demonstrated that unaddressed ‘latent-hidden variables’ during the normalization process can introduce unwanted expression heterogeneity and inadvertent biases [36–38]. These artifacts can subsequently deviate differential expression (DE) analysis downstream and generate higher false positive rates with reduced detection power for true differences [38].

In our study, we determined that normalization by library size was insufficient in removing the high degree of variability to justify a comparative analysis between the two data sets. A preliminary linear regression analysis between our cell lines and TCGA patient tumors, normalized solely by library size, displayed a global shift in higher raw count values for TCGA tumor data in all intra-stage comparisons (Supplementary Figure 1; left panel). These augmented raw count values clearly demonstrated the need for a better normalization method to ensure unbiased expression levels for all subsequent analyses.

Due to the need for better normalization, we utilized another normalization method to mitigate all possible innate biases in our study. The RUVg method is fundamentally a modified version of RUV-2 that adjusts for technical effects as described previously [39–41]. It performs a factor analysis on counts by identifying a set of negative control genes not affected by the biological covariates of interest, but are affected by the factors of unwanted ‘technical’ variation (technical effects are independent of biological conditions of interest). Other researchers that have employed the RUV normalization methods have successfully removed latent variables in bulk RNA-seq experiments [42–47]. In this study, expression variances and overall expression similarities between the two data sets, as demonstrated in the RLE and PCA plots, were markedly improved using RUVg normalization (Figure 1). Furthermore, subsequent DE analysis between stages yielded a much higher number of DEGs (Supplementary Table 2). This increase in sensitivity may be more reflective of the genes regulating changes that may be lost during standard library normalization procedures.

Establishing similarity in overall transcriptome between cells lines and tumors in the TCGA database using the RUVg normalization method allowed us to further investigate signaling pathways and enriched gene sets involved in cancer progression. Expression clustering analysis of DEGs in both instances, stage I and stage II, demonstrated better grouping by stage when compared to advanced stage III; suggesting that the expression profile in advanced stage EC is clearly distinct to early stages (Figure 2A). Furthermore, in contrast to an elevated number of upregulated genes between the early EC stages, a vast majority of genes exhibited downregulation by advanced stage III (Figure 2B). Likewise, prior studies have reported gene downregulation in advanced cancer stages as a consequence of heightened negative feedback in order to maintain network stability despite environmental and genetic stress [48, 49]. Another possibility, as demonstrated in a four-stage model study, indicates that the malignant transformation of tumors may be driven by the downregulation of genes to promote dedifferentiation [50].

In addition to distinguishing the expression pattern of genes between stages in our study, we also identified the top five signaling pathways that may be involved in cancer progression. Three of those pathways specifically the LXR/RXR activation, neuroprotective role for THOP1 in Alzheimer’s disease, and glutamate receptor signaling pathways, shift from being up- to mostly downregulated as approaching advanced cancer stage III. Biological systems rely heavily on negative feedback mechanisms to maintain homeostasis. In signal transduction pathways, feedback inhibition is integral to dampening over activation of signaling output in response to external stimuli or growth factors; however in tumor cells this feedback inhibition is dysregulated by constitutively activated oncoproteins. Moreover, mutational occurrences resulting in the attenuation of negative feedback loop during cancer development may be a critical step in the transformation of tumors to a more aggressive metastatic phenotype [48, 49].

The LXR/RXR pathway is known for regulating cholesterol, glucose, and fatty acid metabolism in a tissue tissue- dependent manner. In addition to its role in metabolism, this pathway is correlated to carcinogenesis [51–58]. Currently, researchers have correlated obesity and elevated cholesterol levels as a major risk factor for malignancy in EC. A study using a luciferase reporter gene system demonstrated that the cholesterol metabolite, 27-hydroxycholesterol (27HC), functions as an agonist for LXR. Stimulation of LXR resulted in the increase of LXR response element (LXRE) transcriptional activity to augment cell proliferation in the Ishikawa cell line [59]. In another study from the endometrium of ovariectomized C57BL/6 mouse given subcutaneous 17-β estradiol (E2) treatment and a high-fat diet (HFD) displayed a divergent action. Their IPA analysis demonstrated a decrease in the LXR/RXR signaling pathway whereas the NF-κB pathway was elevated [60]. Taken together, our findings suggests that an altered LXR/RXR signaling pathway may contribute to the progression of EC and markers identified in this paper should be further investigated.

THOP1 encodes for zinc metalloendopeptidase EC 3.4.24.15 (EP24.15, thimet oligopeptidase), a neuropeptide processing enzyme that is central to the formation and degradation of many bioactive peptides. EP24.15 is also expressed in the periphery and hydrolyzes the neuropeptide, gonadotropin-releasing hormone (GnRH), to yield a biologically active metabolite GnRH-(1–5) [61–66]. The decapeptide GnRH and GnRH analogs have demonstrated to exert an anti-tumorgenic effect in EC [67–70]. However, its metabolite GnRH-(1–5), displays a divergent mechanism of action and biological behavior from its parental peptide [71–73]. In earlier studies, GnRH-(1–5) mediated changes in GnRH-II expression and increased cell proliferation in the Ishikawa cell line [72, 73]. The mechanism for driving cell proliferation and enhanced migration by GnRH(1–5) is through its ability to stimulate the release of epidermal growth factor (EGF) through G protein-coupled receptor 101 (GPR101) to activate the EGF receptor (EGFR) signaling pathway [74]. A subsequent study identified EGF release and increased cellular invasion to be dependent on matrix metallopeptidase (MMP)-9 activity; suggesting the possibility of its role in increasing cellular metastatic potential [75]. Future studies should address the relationship between increased THOP1 expression and enzymatic activity to all its related markers identified in this paper to ascertain its role in driving cancer progression.

Glutamate is a primary excitatory neurotransmitter in the CNS. It has also been implicated in exerting proliferative effects on peripheral tumors through its behavior as a growth factor and subsequent activation of known oncogenic signaling pathways [76–79]. Recent studies have suggested that the altered expression of specific glutamate receptor subunits in cancer cells may regulate DNA repair and intracellular signaling. As a consequence, the stimulation of angiogenesis and cell proliferation leads to the promotion of malignant phenotype and metastatic potential [76, 77, 79]. All things considered, while these observations are intriguing, the biological function or purpose for the shift in expression of genes identified between stages still remains elusive and warrants further investigation.

In addition to signaling pathways, we also identified enriched gene sets to define stage-dependent changes correlated to biological relevance. As seen previously with the signaling transduction analyses, enriched gene sets involving neurogenesis and neuron part emerges several times between stage comparisons and may be a potential driver for metastatic transition across all cancer stages (Figure 3C and 3D). Abnormal neuronal growth and innervation within the endometrium has been correlated to infertility, uterine dysfunction, and endometriosis [80–86]. Previous studies have noted associations between the nervous system and cancer by implicating nerves as having an important role in tumor growth, invasion, and metastasis [87, 88]. Autonomic nerves, specifically the sympathetic nerves, demonstrated a significant role in progression of prostate, gastric, and breast cancers by regulating the cancer microenvironment and immune checkpoints [89–91]. Therefore targeting cancer neurogenesis with corresponding neuronal markers with possible autocrine function may be a promising development in new cancer treatment.

In conclusion, the conventional method of staging classifications to define patients groups to standardize management has been limited due to inconsistencies in tumor behavior, heterogeneity, ambiguous histology, and overlapping molecular characteristics. Here we demonstrate that with the appropriate normalization, we were able to correlate progression in histological staging with transcriptomics that is conserved in both cells lines and TCGA patient tumor sets. The signaling pathways and markers identified in this paper may possibly be used to define and distinguish molecular changes between stages. We demonstrate a substantial down-regulation of genes between early and advanced staged tumors with an altered expression pattern of neuronal signaling pathways and markers. These findings may serve as a novel and promising development in the cancer field as the initial function in these neuronal markers may have a different role and function in the periphery.

## MATERIALS AND METHODS

### Cell culture

The human endometrial adenocarcinoma cell line, the Ishikawa cell line [92], was obtained from American Type Culture Collection (ATCC) (Manassas, VA) [93]. The primary tumor-derived endometrial adenocarcinoma cell lines originated from patients with Stage IC Grade 3 (ACI-181), Stage IIB Grade 2 (ACI-52), and Stage IIIC Grade 2 (ACI-80) International Federation of Gynecology and Obstetrics (FIGO) staging (gift from Dr. Risinger, Michigan State University, Department of Obstetrics, Gynecology and Reproductive Biology, Michigan State University, Grand Rapids 49503, MI, USA) (Supplementary Table 1). All cell lines used in this study are identified as having endometrioid histologic characteristics and were grown-maintained as previously described [74, 75]. In brief, cells were grown in phenol red free-DMEM (Cellgro-Mediatech, Inc., Manassas, VA, USA) supplemented with 10% FBS (Atlanta Biologicals, Lawrenceville, GA, USA) and 2 mM L-Glutamine (Quality Biological Inc., Gaithersburg, MD, USA). These cells were maintained at 37°C with humidified atmosphere of 5% CO_2_ until 90–100% confluence was reached. Cells were subsequently passaged in a 1:5 ratio into 10-cm dishes (Costar, Corning, NY, USA).

### RNA extraction and data acquisition

Total RNA was extracted using Trizol reagent (Invitrogen, Carlsbad, CA, USA) according to manufacturer’s recommendations then purified with DNase I using RNeasy Mini Kit (Qiagen, Germantown, MD). Sequencing libraries were generated from purified RNA from cell lines as described previously [94, 95]. Illumina reads in FASTQ format were trimmed and cropped using Trimmomatic before aligning and mapping to Genome Reference Consortium Human Build 38 patch release 7 (GRCh38.p7) using HISAT Alignment v2.0. Subsequent processing with Samtools v1.3.1 and HTSeq 0.6.0 generated counts based on the number of reads that matched each gene in an annotation file in gene transfer format (GTF). All raw RNA-Seq data for the primary tumor-derived EC cell lines discussed in this publication have been submitted to the SRA database under the accession number SRP074707, and BioProject accession number PRJNA321028. RNA-Seq data acquisition from patients with similar histology, staging, and grading to the primary tumor-derived cell lines were obtained from TCGA cBio Cancer Genomics Portal (http://www.cbioportal.org) in HTSeq file format. Count tables generated by HTSeq-count were imported into R version 3.5.1.

### Normalization methods and assessment of data variation

Previous studies have demonstrated that normalization in RNA-seq data is a crucial step to consider due to its impact on DEGs downstream [35–38, 96, 97]. Numerous factors in our study may introduce nuisance technical effects (i.e., multiple sequencing centers, low input, differences in sequencing depth, gene length biases, varying library kits, flow cells, batches, different experimenters) leading to unwanted bias in our expression sets [36, 40, 96]. Here we employed two normalization methods and determined which was most suitable approach under these experimental conditions. Normalization methods on raw counts using library size or RUVg method were processed as previously described [39–42]. The RUVg method, in brief, utilizes factor analysis to adjust counts for unwanted technical effects based on negative control genes that is determined a priori, *in silico*, which are not affected by the biological covariates of interest. The observed read counts are regressed on both the known covariates of interest and unknown nuisance variables (factors of unwanted variation, k). Although there is no clear cut way for determining k, the number of factors of unwanted variation, k = 3, for this study was selected by considering sample size (number of DEGs obtained) and the degree of technical effects (represented by error bar magnitude) demonstrated by varying k values [39–42](Figure 1A).

For a preliminary determination of whether the global transcriptome of TCGA patients and cell lines are comparative, the counts per million (CPM) of each gene was log transformed to log_2_CPM. Each gene was plotted for TCGA patients vs. cell lines for each normalization method. The R^2^ values were assessed to determine how similar TCGA tumor samples were to cell lines. Scatter plots and R^2^ values were generated using SigmaPlot 10.0.

The effectiveness of normalization in removing variability and improving clustering between samples was assessed using RLE and PCA. RLE is a diagnostic box plot that is useful in visually presenting overall quality and distributions of transformed read counts of each gene across samples. The distribution of the log-ratio of a read count of each gene to the median count across samples that have unwanted variation removed should be centered at the zero line. Furthermore, comparable samples should display similar RLE distributions. The PCA plot displays clusters of samples by assessing similarities in overall gene expression [97–99]. It also describes variation and accounts for varied influences of the original characteristics. The principal components are orthogonal linear combinations of gene expression profiles for each sample. Similarly expressed groups will cluster by class in the first few PCs. Clustering will also highlight possible batch effects and outlying samples. The RLE and PCA analysis were performed using EDASeq packages in R [40].

### Differential gene expression analysis and clustering

DEGs between each stage comparisons in cell lines and tumors were determined by negative binomial generalized linear models (GLMs) by weighted likelihood empirical Bayes with estimate dispersion within edgeR [35]. Genes with false discovery rate (FDR) < 0.05 were considered differentially expressed. To ascertain whether various normalization methods have an impact on downstream differential expression results, we considered changes in the number of DEGs obtained. Once a normalization method was selected, hierarchical heat maps of DEGs and respective volcano plots were generated to determine clustering of stage comparisons-sample types and to identify sets of up- and downregulated DEGs with each stage comparison (*p* < 0.05 and |log_2_FC| > 1). *P*-values instead of FDR values were used for all downstream bioinformatics analysis for statistical uniformity unless indicated. Differential gene expression analysis, heat maps, and volcano plots were performed using gplot function and packages edgeR [35], RUVSeq [40], EDASeq [40], ggplot2 [100], and Rcpp [101] in R environment.

### Ingenuity pathway analysis and gene ontology analysis

The Core analysis feature of the IPA software (Ingenuity Systems, https://www.ingenuity.com [Qiagen]) was used to discover signaling pathways that may regulate cancer progression during stage comparison analysis. The list of DEGs for each stage comparisons were uploaded and categorized to related canonical pathways based on the IPKB. This analysis was set to include direct and indirect relationships and filtered to only consider molecules and/or relationships of the human species. Cutoffs for gene inputs were set to *p* < 0.05 and |log_2_FC| > 1 for down-and upregulated gene expression. Pathways with an overlapping *p*-value < 0.05 calculated by Fisher’s exact test right tailed were considered to be significant. The z-score as indicated by the color intensity considers the match between expected relationship direction (gene expression from IPKB) and observed gene expression. Only z-scores <-2 or >2 were considered significant. The identification of gene sets that define stage-dependent changes or are conserved across all stage comparisons were depicted using Venn diagrams. Only DEGs that were positively mapped ID in the IPA analyses with a *p* < 0.05 and |log_2_FC| > 1 were considered. The GSEA analyses using the Molecular Signatures Databases (MSigDB v6.2) on each venn zone was performed using tools from GSEA Broad Institute (http://software.broadinstitute.org/gsea/msigdb/annotate.jsp) [102–104]. Gene sets corresponding to GO terms with FDR *q*-value < 0.05 were considered significantly enriched. Venn diagrams were performed using VennDiagram package in R.

## SUPPLEMENTARY MATERIALS









## Abbreviations

EC: endometrial cancer
TCGA: The Cancer Genome Atlas
CNS: central nervous system
SEER: Surveillance Epidemiology, and End Results
OS: overall survival rate
POLE: polymerase epsilon
FIGO: International Federation of Gynecology and Obstetrics
RUVg: removal of unwanted variation by control genes
RLE: relative log expression
PCA: principal component analysis
DEG: differentially expressed genes
IPA: Ingenuity Pathway Analysis
LXR/RXR: liver X receptor/retinoid X receptor
LXRE: LXR response element
IPKB: Ingenuity Pathway Knowledge Base
GSEA: gene set enrichment analysis
GO: gene ontology
DE: differential expression
EP24.15: zinc metalloendopeptidase EC 3.4.24.15
GnRH: gonadotropin-releasing hormone
EGF: epidermal growth factor
GPR101: G protein-coupled receptor 101 (GPR101)
EGFR: EGF receptor
MMP-9: matrix metallopeptidase (MMP)-9 activity

## References

[1] Society AC . Cancer Facts and Figures 2021. American Cancer Society: Atlanta, GA. 2021.

[2] Moore K , Brewer MA . Endometrial Cancer: Is This a New Disease? Am Soc Clin Oncol Educ Book. 2017; 37:435–42. 10.1200/EDBK_175666. 28561715

[3] Lee YC , Lheureux S , Oza AM . Treatment strategies for endometrial cancer: current practice and perspective. Curr Opin Obstet Gynecol. 2017; 29:47–58. 10.1097/GCO.0000000000000338. 27941361

[4] Busch EL , Crous-Bou M , Prescott J , Chen MM , Downing MJ , Rosner BA , Mutter GL , De Vivo I . Endometrial Cancer Risk Factors, Hormone Receptors, and Mortality Prediction. Cancer Epidemiol Biomarkers Prev. 2017; 26:727–35. 10.1158/1055-9965.EPI-16-0821. 28052940 PMC5413416

[5] Kaaks R , Lukanova A , Kurzer MS . Obesity, endogenous hormones, and endometrial cancer risk: a synthetic review. Cancer Epidemiol Biomarkers Prev. 2002; 11:1531–43. 12496040

[6] Siegel RL , Miller KD , Jemal A . Cancer statistics, 2019. CA Cancer J Clin. 2019; 69:7–34. 10.3322/caac.21551. 30620402

[7] Colombo N , Preti E , Landoni F , Carinelli S , Colombo A , Marini C , Sessa C , and ESMO Guidelines Working Group. Endometrial cancer: ESMO Clinical Practice Guidelines for diagnosis, treatment and follow-up. Ann Oncol. 2013; 24:vi33–38. 10.1093/annonc/mdt353. 24078661

[8] Brinton LA , Felix AS , McMeekin DS , Creasman WT , Sherman ME , Mutch D , Cohn DE , Walker JL , Moore RG , Downs LS , Soslow RA , Zaino R . Etiologic heterogeneity in endometrial cancer: evidence from a Gynecologic Oncology Group trial. Gynecol Oncol. 2013; 129:277–84. 10.1016/j.ygyno.2013.02.023. 23485770 PMC4006113

[9] Voss MA , Ganesan R , Ludeman L , McCarthy K , Gornall R , Schaller G , Wei W , Sundar S . Should grade 3 endometrioid endometrial carcinoma be considered a type 2 cancer-a clinical and pathological evaluation. Gynecol Oncol. 2012; 124:15–20. 10.1016/j.ygyno.2011.07.030. 21864888

[10] Hussein YR , Broaddus R , Weigelt B , Levine DA , Soslow RA . The Genomic Heterogeneity of FIGO Grade 3 Endometrioid Carcinoma Impacts Diagnostic Accuracy and Reproducibility. Int J Gynecol Pathol. 2016; 35:16–24. 10.1097/PGP.0000000000000212. 26166718 PMC4934379

[11] Bendifallah S , Canlorbe G , Collinet P , Arsène E , Huguet F , Coutant C , Hudry D , Graesslin O , Raimond E , Touboul C , Daraï E , Ballester M . Just how accurate are the major risk stratification systems for early-stage endometrial cancer? Br J Cancer. 2015; 112:793–801. 10.1038/bjc.2015.35. 25675149 PMC4453957

[12] Arend RC , Jones BA , Martinez A , Goodfellow P . Endometrial cancer: Molecular markers and management of advanced stage disease. Gynecol Oncol. 2018; 150:569–80. 10.1016/j.ygyno.2018.05.015. 29843906

[13] Llauradó M , Ruiz A , Majem B , Ertekin T , Colás E , Pedrola N , Devis L , Rigau M , Sequeiros T , Montes M , Garcia M , Cabrera S , Gil-Moreno A , et al. Molecular bases of endometrial cancer: new roles for new actors in the diagnosis and the therapy of the disease. Mol Cell Endocrinol. 2012; 358:244–55. 10.1016/j.mce.2011.10.003. 22037169

[14] Pandita P , Wang X , Jones DE , Collins K , Hawkins SM . Unique Molecular Features in High-Risk Histology Endometrial Cancers. Cancers (Basel). 2019; 11:1665. 10.3390/cancers11111665. 31717878 PMC6896116

[15] Murali R , Soslow RA , Weigelt B . Classification of endometrial carcinoma: more than two types. Lancet Oncol. 2014; 15:e268–78. 10.1016/S1470-2045(13)70591-6. 24872110

[16] Suarez AA , Felix AS , Cohn DE . Bokhman Redux: Endometrial cancer "types" in the 21st century. Gynecol Oncol. 2017; 144:243–49. 10.1016/j.ygyno.2016.12.010. 27993480

[17] Goodspeed A , Heiser LM , Gray JW , Costello JC . Tumor-Derived Cell Lines as Molecular Models of Cancer Pharmacogenomics. Mol Cancer Res. 2016; 14:3–13. 10.1158/1541-7786.MCR-15-0189. 26248648 PMC4828339

[18] Ertel A , Verghese A , Byers SW , Ochs M , Tozeren A . Pathway-specific differences between tumor cell lines and normal and tumor tissue cells. Mol Cancer. 2006; 5:55. 10.1186/1476-4598-5-55. 17081305 PMC1635729

[19] Bild AH , Yao G , Chang JT , Wang Q , Potti A , Chasse D , Joshi MB , Harpole D , Lancaster JM , Berchuck A , Olson JA Jr , Marks JR , Dressman HK , et al. Oncogenic pathway signatures in human cancers as a guide to targeted therapies. Nature. 2006; 439:353–57. 10.1038/nature04296. 16273092

[20] Gillet JP , Calcagno AM , Varma S , Marino M , Green LJ , Vora MI , Patel C , Orina JN , Eliseeva TA , Singal V , Padmanabhan R , Davidson B , Ganapathi R , et al. Redefining the relevance of established cancer cell lines to the study of mechanisms of clinical anti-cancer drug resistance. Proc Natl Acad Sci U S A. 2011; 108:18708–13. 10.1073/pnas.1111840108. 22068913 PMC3219108

[21] Domcke S , Sinha R , Levine DA , Sander C , Schultz N . Evaluating cell lines as tumour models by comparison of genomic profiles. Nat Commun. 2013; 4:2126. 10.1038/ncomms3126. 23839242 PMC3715866

[22] Chandrani P , Upadhyay P , Iyer P , Tanna M , Shetty M , Raghuram GV , Oak N , Singh A , Chaubal R , Ramteke M , Gupta S , Dutt A . Integrated genomics approach to identify biologically relevant alterations in fewer samples. BMC Genomics. 2015; 16:936. 10.1186/s12864-015-2138-4. 26572163 PMC4647579

[23] Yu K , Chen B , Aran D , Charalel J , Yau C , Wolf DM , van’t Veer LJ , Butte AJ , Goldstein T , Sirota M . Comprehensive transcriptomic analysis of cell lines as models of primary tumors across 22 tumor types. Nat Commun. 2019; 10:3574. 10.1038/s41467-019-11415-2. 31395879 PMC6687785

[24] Kandoth C , Schultz N , Cherniack AD , Akbani R , Liu Y , Shen H , Robertson AG , Pashtan I , Shen R , Benz CC , Yau C , Laird PW , Ding L , et al, and Cancer Genome Atlas Research Network. Integrated genomic characterization of endometrial carcinoma. Nature. 2013; 497:67–73. 10.1038/nature12113. 23636398 PMC3704730

[25] Liang H , Cheung LW , Li J , Ju Z , Yu S , Stemke-Hale K , Dogruluk T , Lu Y , Liu X , Gu C , Guo W , Scherer SE , Carter H , et al. Whole-exome sequencing combined with functional genomics reveals novel candidate driver cancer genes in endometrial cancer. Genome Res. 2012; 22:2120–29. 10.1101/gr.137596.112. 23028188 PMC3483541

[26] Kuhn E , Ayhan A , Bahadirli-Talbott A , Zhao C , Shih IM . Molecular characterization of undifferentiated carcinoma associated with endometrioid carcinoma. Am J Surg Pathol. 2014; 38:660–65. 10.1097/PAS.0000000000000166. 24451280

[27] Talhouk A , McConechy MK , Leung S , Li-Chang HH , Kwon JS , Melnyk N , Yang W , Senz J , Boyd N , Karnezis AN , Huntsman DG , Gilks CB , McAlpine JN . A clinically applicable molecular-based classification for endometrial cancers. Br J Cancer. 2015; 113:299–310. 10.1038/bjc.2015.190. 26172027 PMC4506381

[28] Salvesen HB , Carter SL , Mannelqvist M , Dutt A , Getz G , Stefansson IM , Raeder MB , Sos ML , Engelsen IB , Trovik J , Wik E , Greulich H , Bø TH , et al. Integrated genomic profiling of endometrial carcinoma associates aggressive tumors with indicators of PI3 kinase activation. Proc Natl Acad Sci U S A. 2009; 106:4834–39. 10.1073/pnas.0806514106. 19261849 PMC2660768

[29] Diver EJ , Foster R , Rueda BR , Growdon WB . The Therapeutic Challenge of Targeting HER2 in Endometrial Cancer. Oncologist. 2015; 20:1058–68. 10.1634/theoncologist.2015-0149. 26099744 PMC4571805

[30] Charo LM , Plaxe SC . Recent advances in endometrial cancer: a review of key clinical trials from 2015 to 2019. F1000Res. 2019; 8:F1000. 10.12688/f1000research.17408.1. 31231511 PMC6567288

[31] Liu Y , Broaddus RR , Zhang W . Identifying aggressive forms of endometrioid-type endometrial cancer: new insights into molecular subtyping. Expert Rev Anticancer Ther. 2015; 15:1–3. 10.1586/14737140.2015.992420. 25494844 PMC4638381

[32] Hong B , Le Gallo M , Bell DW . The mutational landscape of endometrial cancer. Curr Opin Genet Dev. 2015; 30:25–31. 10.1016/j.gde.2014.12.004. 25622247 PMC4476916

[33] Imboden S , Nastic D , Ghaderi M , Rydberg F , Rau TT , Mueller MD , Epstein E , Carlson JW . Phenotype of POLE-mutated endometrial cancer. PLoS One. 2019; 14:e0214318. 10.1371/journal.pone.0214318. 30917185 PMC6436745

[34] Adishesh M , Hapangama DK . Enriching Personalized Endometrial Cancer Research with the Harmonization of Biobanking Standards. Cancers (Basel). 2019; 11:1734. 10.3390/cancers11111734. 31694311 PMC6896027

[35] Robinson MD , Oshlack A . A scaling normalization method for differential expression analysis of RNA-seq data. Genome Biol. 2010; 11:R25. 10.1186/gb-2010-11-3-r25. 20196867 PMC2864565

[36] Bullard JH , Purdom E , Hansen KD , Dudoit S . Evaluation of statistical methods for normalization and differential expression in mRNA-Seq experiments. BMC Bioinformatics. 2010; 11:94. 10.1186/1471-2105-11-94. 20167110 PMC2838869

[37] Evans C , Hardin J , Stoebel DM . Selecting between-sample RNA-Seq normalization methods from the perspective of their assumptions. Brief Bioinform. 2018; 19:776–92. 10.1093/bib/bbx008. 28334202 PMC6171491

[38] Dillies MA , Rau A , Aubert J , Hennequet-Antier C , Jeanmougin M , Servant N , Keime C , Marot G , Castel D , Estelle J , Guernec G , Jagla B , Jouneau L , et al, and French StatOmique Consortium. A comprehensive evaluation of normalization methods for Illumina high-throughput RNA sequencing data analysis. Brief Bioinform. 2013; 14:671–83. 10.1093/bib/bbs046. 22988256

[39] Gagnon-Bartsch JA , Speed TP . Using control genes to correct for unwanted variation in microarray data. Biostatistics. 2012; 13:539–52. 10.1093/biostatistics/kxr034. 22101192 PMC3577104

[40] Risso D , Ngai J , Speed TP , Dudoit S . Normalization of RNA-seq data using factor analysis of control genes or samples. Nat Biotechnol. 2014; 32:896–902. 10.1038/nbt.2931. 25150836 PMC4404308

[41] Maksimovic J , Gagnon-Bartsch JA , Speed TP , Oshlack A . Removing unwanted variation in a differential methylation analysis of Illumina HumanMethylation450 array data. Nucleic Acids Res. 2015; 43:e106. 10.1093/nar/gkv526. 25990733 PMC4652745

[42] Westermann AJ , Förstner KU , Amman F , Barquist L , Chao Y , Schulte LN , Müller L , Reinhardt R , Stadler PF , Vogel J . Dual RNA-seq unveils noncoding RNA functions in host-pathogen interactions. Nature. 2016; 529:496–501. 10.1038/nature16547. 26789254

[43] Kawakatsu T , Huang SC , Jupe F , Sasaki E , Schmitz RJ , Urich MA , Castanon R , Nery JR , Barragan C , He Y , Chen H , Dubin M , Lee CR , et al. Epigenomic Diversity in a Global Collection of Arabidopsis thaliana Accessions. Cell. 2016; 166:492–505. 10.1016/j.cell.2016.06.044. 27419873 PMC5172462

[44] Labonté B , Engmann O , Purushothaman I , Menard C , Wang J , Tan C , Scarpa JR , Moy G , Loh YE , Cahill M , Lorsch ZS , Hamilton PJ , Calipari ES , et al. Sex-specific transcriptional signatures in human depression. Nat Med. 2017; 23:1102–11. 10.1038/nm.4386. 28825715 PMC5734943

[45] Fogarty NME , McCarthy A , Snijders KE , Powell BE , Kubikova N , Blakeley P , Lea R , Elder K , Wamaitha SE , Kim D , Maciulyte V , Kleinjung J , Kim JS , et al. Genome editing reveals a role for OCT4 in human embryogenesis. Nature. 2017; 550:67–73. 10.1038/nature24033. 28953884 PMC5815497

[46] Chrispijn ND , Elurbe DM , Mickoleit M , Aben M , de Bakker DEM , Andralojc KM , Huisken J , Bakkers J , Kamminga LM . Loss of the Polycomb group protein Rnf2 results in derepression of tbx-transcription factors and defects in embryonic and cardiac development. Sci Rep. 2019; 9:4327. 10.1038/s41598-019-40867-1. 30867528 PMC6416260

[47] Wendorff AA , Quinn SA , Rashkovan M , Madubata CJ , Ambesi-Impiombato A , Litzow MR , Tallman MS , Paietta E , Paganin M , Basso G , Gastier-Foster JM , Loh ML , Rabadan R , et al. Phf6 Loss Enhances HSC Self-Renewal Driving Tumor Initiation and Leukemia Stem Cell Activity in T-ALL. Cancer Discov. 2019; 9:436–51. 10.1158/2159-8290.CD-18-1005. 30567843 PMC6425751

[48] Chandarlapaty S . Negative feedback and adaptive resistance to the targeted therapy of cancer. Cancer Discov. 2012; 2:311–19. 10.1158/2159-8290.CD-12-0018. 22576208 PMC3351275

[49] Foulds CE . Disrupting a negative feedback loop drives endocrine therapy-resistant breast cancer. Proc Natl Acad Sci U S A. 2018; 115:8236–38. 10.1073/pnas.1811263115. 30082387 PMC6099889

[50] Danielsson F , Skogs M , Huss M , Rexhepaj E , O’Hurley G , Klevebring D , Pontén F , Gad AK , Uhlén M , Lundberg E . Majority of differentially expressed genes are down-regulated during malignant transformation in a four-stage model. Proc Natl Acad Sci U S A. 2013; 110:6853–58. 10.1073/pnas.1216436110. 23569271 PMC3637701

[51] Lin CY , Gustafsson JÅ . Targeting liver X receptors in cancer therapeutics. Nat Rev Cancer. 2015; 15:216–24. 10.1038/nrc3912. 25786697

[52] Lin EW , Karakasheva TA , Lee DJ , Lee JS , Long Q , Bass AJ , Wong KK , Rustgi AK . Comparative transcriptomes of adenocarcinomas and squamous cell carcinomas reveal molecular similarities that span classical anatomic boundaries. PLoS Genet. 2017; 13:e1006938. 10.1371/journal.pgen.1006938. 28787442 PMC5560753

[53] Torres-Luquis O , Madden K , N’dri NM , Berg R , Olopade OF , Ngwa W , Abuidris D , Mittal S , Lyn-Cook B , Mohammed SI . LXR/RXR pathway signaling associated with triple-negative breast cancer in African American women. Breast Cancer (Dove Med Press). 2019; 11:1–12. 10.2147/BCTT.S185960. 30588086 PMC6304259

[54] Villablanca EJ , Raccosta L , Zhou D , Fontana R , Maggioni D , Negro A , Sanvito F , Ponzoni M , Valentinis B , Bregni M , Prinetti A , Steffensen KR , Sonnino S , et al. Tumor-mediated liver X receptor-alpha activation inhibits CC chemokine receptor-7 expression on dendritic cells and dampens antitumor responses. Nat Med. 2010; 16:98–105. 10.1038/nm.2074. 20037595

[55] Dai YB , Miao YF , Wu WF , Li Y , D’Errico F , Su W , Burns AR , Huang B , Maneix L , Warner M , Gustafsson JÅ . Ablation of Liver X receptors α and β leads to spontaneous peripheral squamous cell lung cancer in mice. Proc Natl Acad Sci U S A. 2016; 113:7614–19. 10.1073/pnas.1607590113. 27335465 PMC4941512

[56] El Roz A , Bard JM , Huvelin JM , Nazih H . LXR agonists and ABCG1-dependent cholesterol efflux in MCF-7 breast cancer cells: relation to proliferation and apoptosis. Anticancer Res. 2012; 32:3007–13. 22753765

[57] Flaveny CA , Griffett K , El-Gendy BEM , Kazantzis M , Sengupta M , Amelio AL , Chatterjee A , Walker J , Solt LA , Kamenecka TM , Burris TP . Broad Anti-tumor Activity of a Small Molecule that Selectively Targets the Warburg Effect and Lipogenesis. Cancer Cell. 2015; 28:42–56. 10.1016/j.ccell.2015.05.007. 26120082 PMC4965273

[58] Bovenga F , Sabbà C , Moschetta A . Uncoupling nuclear receptor LXR and cholesterol metabolism in cancer. Cell Metab. 2015; 21:517–26. 10.1016/j.cmet.2015.03.002. 25863245

[59] Gibson DA , Collins F , Cousins FL , Esnal Zufiaurre A , Saunders PTK . The impact of 27-hydroxycholesterol on endometrial cancer proliferation. Endocr Relat Cancer. 2018; 25:381–91. 10.1530/ERC-17-0449. 29371332 PMC5847183

[60] Cheng Y , Lv Q , Xie B , Yang B , Shan W , Ning C , Li B , Xie L , Gu C , Luo X , Chen X , Zhu Q . Estrogen and high-fat diet induced alterations in C57BL/6 mice endometrial transcriptome profile. Endocr Connect. 2018; 7:36–46. 10.1530/EC-17-0315. 29133384 PMC5744625

[61] Swanson TA , Kim SI , Myers M , Pabon A , Philibert KD , Wang M , Glucksman MJ . The role of neuropeptide processing enzymes in endocrine (prostate) cancer: EC 3.4.24.15 (EP24.15). Protein Pept Lett. 2004; 11:471–78. 10.2174/0929866043406607. 15544568

[62] Maggi R , Moretti R , Marelli M , Pimpinelli F , Motta M . Human prostatic-carcinoma cell-line lncap degrades luteinizing-hormone-releasing hormone. Int J Oncol. 1995; 6:1231–36. 10.3892/ijo.6.6.1231. 21556662

[63] Oliveira V , Garrido PA , Rodrigues CC , Colquhoun A , Castro LM , Almeida PC , Shida CS , Juliano MA , Juliano L , Camargo AC , Hyslop S , Roberts JL , Grum-Tokars V , et al. Calcium modulates endopeptidase 24.15 (EC 3.4.24.15) membrane association, secondary structure and substrate specificity. FEBS J. 2005; 272:2978–92. 10.1111/j.1742-4658.2005.04692.x. 15955058

[64] Ribeiro DA , Nascimento FD , Fracalossi AC , Noguti J , Oshima CT , Ihara SS , Franco MF . The role of metalloendopeptidases in oropharyngeal carcinomas assessed by tissue microarray. Cancer Genomics Proteomics. 2011; 8:307–10. 22086898

[65] Ferro ES , Tullai JW , Glucksman MJ , Roberts JL . Secretion of metalloendopeptidase 24.15 (EC 3.4.24.15). DNA Cell Biol. 1999; 18:781–89. 10.1089/104454999314926. 10541437

[66] Roberts JL , Mani SK , Woller MJ , Glucksman MJ , Wu TJ . LHRH-(1-5): a bioactive peptide regulating reproduction. Trends Endocrinol Metab. 2007; 18:386–92. 10.1016/j.tem.2007.09.005. 17997103

[67] Gründker C , Günthert AR , Westphalen S , Emons G . Biology of the gonadotropin-releasing hormone system in gynecological cancers. Eur J Endocrinol. 2002; 146:1–14. 10.1530/eje.0.1460001. 11751060

[68] Emons G , Ortmann O , Schulz KD , Schally AV . Growth-inhibitory actions of analogues of Luteinizing Hormone Releasing Hormone on tumor cells. Trends Endocrinol Metab. 1997; 8:355–62. 10.1016/s1043-2760(97)00155-0. 18406825

[69] Gallagher CJ , Oliver RT , Oram DH , Fowler CG , Blake PR , Mantell BS , Slevin ML , Hope-Stone HF . A new treatment for endometrial cancer with gonadotrophin releasing-hormone analogue. Br J Obstet Gynaecol. 1991; 98:1037–41. 10.1111/j.1471-0528.1991.tb15343.x. 1751436

[70] Kullander S . Treatment of endometrial cancer with GnRH analogs. Recent Results Cancer Res. 1992; 124:69–73. 10.1007/978-88-470-2186-0_7. 1615220

[71] Wu TJ , Glucksman MJ , Roberts JL , Mani SK . Facilitation of lordosis in rats by a metabolite of luteinizing hormone releasing hormone. Endocrinology. 2006; 147:2544–49. 10.1210/en.2005-1646. 16497796

[72] Walters K , Chin YP , Wu TJ . A processed metabolite of luteinizing hormone-releasing hormone has proliferative effects in endometrial cells. Am J Obstet Gynecol. 2007; 196:33.e1–5. 10.1016/j.ajog.2006.07.054. 17240223

[73] Baldwin EL , Wegorzewska IN , Flora M , Wu TJ . Regulation of type II luteinizing hormone-releasing hormone (LHRH-II) gene expression by the processed peptide of LHRH-I, LHRH-(1-5) in endometrial cells. Exp Biol Med (Maywood). 2007; 232:146–55. 17202595

[74] Cho-Clark M , Larco DO , Semsarzadeh NN , Vasta F , Mani SK , Wu TJ . GnRH-(1-5) transactivates EGFR in Ishikawa human endometrial cells via an orphan G protein-coupled receptor. Mol Endocrinol. 2014; 28:80–98. 10.1210/me.2013-1203. 24264576 PMC5426651

[75] Cho-Clark M , Larco DO , Zahn BR , Mani SK , Wu TJ . GnRH-(1-5) activates matrix metallopeptidase-9 to release epidermal growth factor and promote cellular invasion. Mol Cell Endocrinol. 2015; 415:114–25. 10.1016/j.mce.2015.08.010. 26277400

[76] Stepulak A , Rola R , Polberg K , Ikonomidou C . Glutamate and its receptors in cancer. J Neural Transm (Vienna). 2014; 121:933–44. 10.1007/s00702-014-1182-6. 24610491 PMC4133641

[77] Haas HS , Pfragner R , Tabrizi-Wizsy NG , Rohrer K , Lueftenegger I , Horwath C , Allard N , Rinner B , Sadjak A . The influence of glutamate receptors on proliferation and metabolic cell activity of neuroendocrine tumors. Anticancer Res. 2013; 33:1267–72. 23564764

[78] Hu H , Takano N , Xiang L , Gilkes DM , Luo W , Semenza GL . Hypoxia-inducible factors enhance glutamate signaling in cancer cells. Oncotarget. 2014; 5:8853–68. 10.18632/oncotarget.2593. 25326682 PMC4253402

[79] Luksch H , Uckermann O , Stepulak A , Hendruschk S , Marzahn J , Bastian S , Staufner C , Temme A , Ikonomidou C . Silencing of selected glutamate receptor subunits modulates cancer growth. Anticancer Res. 2011; 31:3181–92. 21965725

[80] Wu J , Xie H , Yao S , Liang Y . Macrophage and nerve interaction in endometriosis. J Neuroinflammation. 2017; 14:53. 10.1186/s12974-017-0828-3. 28288663 PMC5351283

[81] Berkley KJ , Rapkin AJ , Papka RE . The pains of endometriosis. Science. 2005; 308:1587–89. 10.1126/science.1111445. 15947176

[82] Zhang Z , Lei A , Xu L , Chen L , Chen Y , Zhang X , Gao Y , Yang X , Zhang M , Cao Y . Similarity in gene-regulatory networks suggests that cancer cells share characteristics of embryonic neural cells. J Biol Chem. 2017; 292:12842–59. 10.1074/jbc.M117.785865. 28634230 PMC5546026

[83] Newman TA , Bailey JL , Stocker LJ , Woo YL , Macklon NS , Cheong YC . Expression of neuronal markers in the endometrium of women with and those without endometriosis. Hum Reprod. 2013; 28:2502–10. 10.1093/humrep/det274. 23820422

[84] Quinn M . Endometriosis: the consequence of neurological dysfunction? Med Hypotheses. 2004; 63:602–08. 10.1016/j.mehy.2004.03.032. 15325003

[85] Quinn MJ . Endometriosis: the consequence of uterine denervation-reinnervation. Arch Gynecol Obstet. 2011; 284:1423–29. 10.1007/s00404-011-2063-y. 21932088

[86] Quinn MJ , Kirk N . Differences in uterine innervation at hysterectomy. Am J Obstet Gynecol. 2002; 187:1515–19. 10.1067/mob.2002.130007. 12501055

[87] Kuol N , Stojanovska L , Apostolopoulos V , Nurgali K . Role of the nervous system in cancer metastasis. J Exp Clin Cancer Res. 2018; 37:5. 10.1186/s13046-018-0674-x. 29334991 PMC5769535

[88] Li S , Sun Y , Gao D . Role of the nervous system in cancer metastasis. Oncol Lett. 2013; 5:1101–11. 10.3892/ol.2013.1168. 23599747 PMC3629128

[89] Magnon C . Role of the autonomic nervous system in tumorigenesis and metastasis. Mol Cell Oncol. 2015; 2:e975643. 10.4161/23723556.2014.975643. 27308436 PMC4904882

[90] Kamiya A , Hayama Y , Kato S , Shimomura A , Shimomura T , Irie K , Kaneko R , Yanagawa Y , Kobayashi K , Ochiya T . Genetic manipulation of autonomic nerve fiber innervation and activity and its effect on breast cancer progression. Nat Neurosci. 2019; 22:1289–305. 10.1038/s41593-019-0430-3. 31285612

[91] Lu R , Fan C , Shangguan W , Liu Y , Li Y , Shang Y , Yin D , Zhang S , Huang Q , Li X , Meng W , Xu H , Zhou Z , et al. Neurons generated from carcinoma stem cells support cancer progression. Signal Transduct Target Ther. 2017; 2:16036. 10.1038/sigtrans.2016.36. 29263908 PMC5657421

[92] Nishida M . The Ishikawa cells from birth to the present. Hum Cell. 2002; 15:104–17. 10.1111/j.1749-0774.2002.tb00105.x. 12703541

[93] Korch C , Spillman MA , Jackson TA , Jacobsen BM , Murphy SK , Lessey BA , Jordan VC , Bradford AP . DNA profiling analysis of endometrial and ovarian cell lines reveals misidentification, redundancy and contamination. Gynecol Oncol. 2012; 127:241–48. 10.1016/j.ygyno.2012.06.017. 22710073 PMC3432677

[94] Moritz KE , McCormack NM , Abera MB , Viollet C , Yauger YJ , Sukumar G , Dalgard CL , Burnett BG . The role of the immunoproteasome in interferon-γ-mediated microglial activation. Sci Rep. 2017; 7:9365. 10.1038/s41598-017-09715-y. 28839214 PMC5571106

[95] Sudigyo D , Rahmawati G , Setiasari DW , Poluan RH , Sesotyosari SL , Wardana T , Herawati C , Heriyanto DS , Indrasari SR , Afiahayati , Astuti I , Haryana SM . Transcriptome Profile of Next Generation Sequence Data Related to Inflammation on Nasopharyngeal Carcinoma Cases in Indonesia. Asian Pac J Cancer Prev. 2020; 21:2763–69. 10.31557/APJCP.2020.21.9.2763. 32986378 PMC7779428

[96] Rahman M , Jackson LK , Johnson WE , Li DY , Bild AH , Piccolo SR . Alternative preprocessing of RNA-Sequencing data in The Cancer Genome Atlas leads to improved analysis results. Bioinformatics. 2015; 31:3666–72. 10.1093/bioinformatics/btv377. 26209429 PMC4804769

[97] Conesa A , Madrigal P , Tarazona S , Gomez-Cabrero D , Cervera A , McPherson A , Szcześniak MW , Gaffney DJ , Elo LL , Zhang X , Mortazavi A . Erratum to: A survey of best practices for RNA-seq data analysis. Genome Biol. 2016; 17:181. 10.1186/s13059-016-1047-4. 26813401 PMC4728800

[98] Jolliffe IT , Cadima J . Principal component analysis: a review and recent developments. Philos Trans A Math Phys Eng Sci. 2016; 374:20150202. 10.1098/rsta.2015.0202. 26953178 PMC4792409

[99] Son K , Yu S , Shin W , Han K , Kang K . A Simple Guideline to Assess the Characteristics of RNA-Seq Data. Biomed Res Int. 2018; 2018:2906292. 10.1155/2018/2906292. 30519573 PMC6241233

[100] Wickham H . ggplot2: Elegant Graphics for Data Analysis. Springer-Verlag: New York. 2016.

[101] Eddelbuettel D , Francois R . Rcpp: Seamless R and C++ Integration. Journal of Statistical Software. 2011; 40:1–18. 10.18637/jss.v040.i08.

[102] Subramanian A , Tamayo P , Mootha VK , Mukherjee S , Ebert BL , Gillette MA , Paulovich A , Pomeroy SL , Golub TR , Lander ES , Mesirov JP . Gene set enrichment analysis: a knowledge-based approach for interpreting genome-wide expression profiles. Proc Natl Acad Sci U S A. 2005; 102:15545–50. 10.1073/pnas.0506580102. 16199517 PMC1239896

[103] Liberzon A , Subramanian A , Pinchback R , Thorvaldsdóttir H , Tamayo P , Mesirov JP . Molecular signatures database (MSigDB) 3.0. Bioinformatics. 2011; 27:1739–40. 10.1093/bioinformatics/btr260. 21546393 PMC3106198

[104] Liberzon A , Birger C , Thorvaldsdóttir H , Ghandi M , Mesirov JP , Tamayo P . The Molecular Signatures Database (MSigDB) hallmark gene set collection. Cell Syst. 2015; 1:417–25. 10.1016/j.cels.2015.12.004. 26771021 PMC4707969

