# Supplementary material for: Comparative transcriptome analysis between patient and endometrial cancer cell lines to determine common signaling pathways and markers linked to cancer progression
Source: Oncotarget. 2021 Dec 21;12(26):2500–13. doi: 10.18632/oncotarget.28161 (PMC8711572; doi:10.18632/oncotarget.28161)
Supplement: Supplementary file 1 [file oncotarget-12-2500-s001.pdf]

Comparative transcriptome analysis between patient and endometrial cancer cell lines to determine common signaling pathways and markers linked to cancer progression

SUPPLEMENTARY MATERIALS

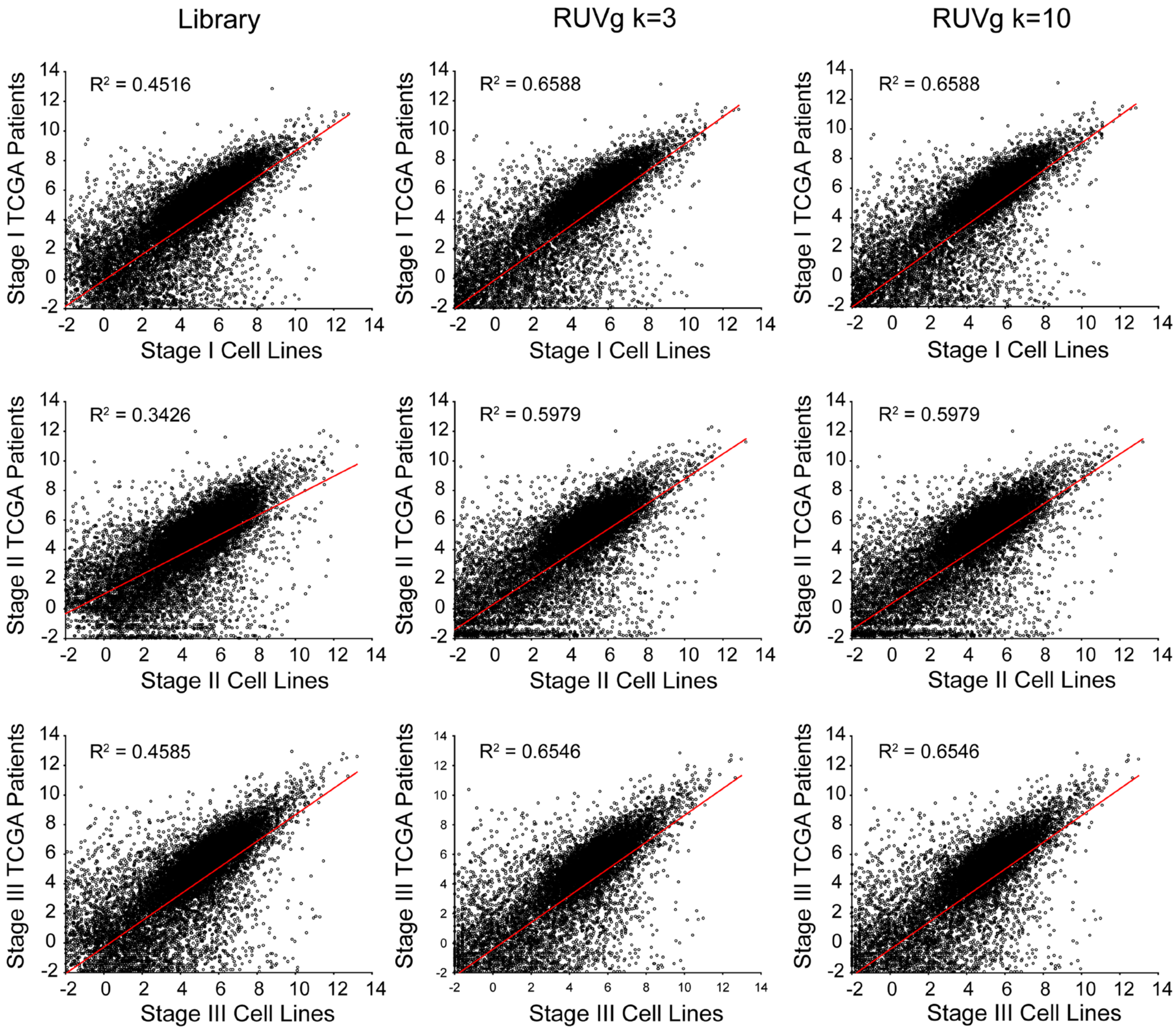

Supplementary Figure 1: Comparison of log_2CPM of genes between TCGA patients (*Y*-axis) and cell lines (*X*-axis). A global shift in higher transcript expression for TCGA patients (genes above the regression line) was demonstrated at each cancer stage.

Supplementary Table 1: Endometrioid adenocarcinoma sample type, number of samples (*n*), name/patient ID, stage, and grade

Sample Type	Number of Samples (*n*)	Name/Patient ID	Stage	Grade
primary tumor-derived cell line	4	ACI-181	IC	III
primary tumor-derived cell line	5	ACI-52	IIB	II
cell line	4	Ishikawa	IIB	II
primary tumor-derived cell line	3	ACI-80	IIIC	II
TCGA tumor	13	TCGA-AX-A060<br>TCGA-BS-A0TJ<br>TCGA-DF-A2KN<br>TCGA-AX-A2IN<br>TCGA-AJ-A3BK<br>TCGA-AX-A2HJ<br>TCGA-B5-A3FA<br>TCGA-AX-A2H7<br>TCGA-AX-A2HG<br>TCGA-BS-A0TI<br>TCGA-D1-A175<br>TCGA-FI-A2F4<br>TCGA-AX-A3G1	IC	III
TCGA tumor	4	TCGA-BS-A0UT<br>TCGA-BS-A0V8<br>TCGA-DI-A1BY<br>TCGA-D1-A16J	IIB	II
TCGA tumor	7	TCGA-D1-A163<br>TCGA-EY-A1GO<br>TCGA-BG-A0M7<br>TCGA-AP-A1DR<br>TCGA-D1-A17U<br>TCGA-AP-A1E0<br>TCGA-EY-A1H0	IIIC	II

Supplementary Table 2: Impact of normalization method and RUVg k value on the number of differentially expressed genes (DEGs) between cancer stages (FDR <0.05)

Normalization Method	Stage I vs. Stage II	Stage II vs. Stage III	Stage I vs. III
library size	2101	1800	355
RUVg k = 3	2905	2466	563
RUVg k = 10	2613	2193	432

Supplementary Table 3: Top 20 gene sets involved in up-or downregulation between cancer stages; significant (FDR <0.05). See Supplementary Table 3

Supplementary Table 4: Ingenuity pathway analysis (Qiagen, Valencia, CA, USA) based on differentially expressed genes between cancer stages (*p*-value <0.05)

Comparison	Top 20 Pathways
Stage I vs. Stage II	LXR/RXR Activation, Neuroprotective Role of THOP1 in Alzheimer's Disease, Glutamate Receptor Signaling, Axonal Guidance Signaling, α-tocopherol Degradation, LPS/IL-1 Mediated Inhibition of RXR Function, Calcium Signaling, Role of Osteoblasts, Osteoclasts and Chondrocytes in Rheumatoid Arthritis, Bupropion Degradation, GP6 Signaling Pathway, Acetone Degradation I (to Methylglyoxal), Melatonin Degradation I, Superpathway of Melatonin Degradation, Amyotrophic Lateral Sclerosis Signaling, Human Embryonic Stem Cell Pluripotency, Transcriptional Regulatory Network in Embryonic Stem Cells, Nicotine Degradation II, Crosstalk between Dendritic Cells and Natural Killer Cells, FXR/RXR Activation, Nicotine Degradation III
Stage II vs. Stage III	Calcium Signaling, nNOS Signaling in Skeletal Muscle Cells, Opioid Signaling Pathway, CREB Signaling in Neurons, Glutamate Receptor Signaling, G Beta Gamma Signaling, GPCR-Mediated Nutrient Sensing in Enteroendocrine Cells, Bladder Cancer Signaling, GABA Receptor Signaling, Synaptic Long Term Depression, Corticotropin Releasing Hormone Signaling, LPS/IL-1 Mediated Inhibition of RXR Function, Role of NFAT in Cardiac Hypertrophy, Axonal Guidance Signaling, Type II Diabetes Mellitus Signaling, Cellular Effects of Sildenafil (Viagra), Inhibition of Matrix Metalloproteases, GNRH Signaling, Gustation Pathway, α-Adrenergic Signaling
Stage I vs. Stage III	nNOS Signaling in Skeletal Muscle Cells, LXR/RXR Activation, FXR/RXR Activation, Neuroprotective Role of THOP1 in Alzheimer's Disease, Calcium Signaling, Intrinsic Prothrombin Activation Pathway, Retinoate Biosynthesis I, LPS/IL-1 Mediated Inhibition of RXR Function, Neuropathic Pain, Signaling In Dorsal Horn Neurons, The Visual Cycle, Atherosclerosis Signaling, MSP-RON Signaling Pathway, Histidine Degradation VI, Opioid Signaling Pathway, Clathrin-mediated Endocytosis Signaling, Coagulation System, Netrin Signaling, GP6 Signaling Pathway, CREB Signaling in Neurons, Retinol Biosynthesis

Supplementary Table 5: Differentially expressed genes in the top 5 signaling pathways between cancer stages. See Supplementary Table 5

Supplementary Table 6: Notable biological processes involved in each zone of Venn diagram; significant (FDR <0.05). See Supplementary Table 6
