# Supplementary material for: Comparative transcriptome analysis between patient and endometrial cancer cell lines to determine common signaling pathways and markers linked to cancer progression
Source: Oncotarget. 2021 Dec 21;12(26):2500–13. doi: 10.18632/oncotarget.28161 (PMC8711572; doi:10.18632/oncotarget.28161)
Supplement: Supplementary file 2 [file oncotarget-12-2500-s002.docx]

**Supplementary Table 3: Top 20 gene sets involved in up-or downregulation between cancer stages; significant (FDR <0.05)**

| Comparisons |  | Gene Set Name | # of Genes | FDR  q-value |
| --- | --- | --- | --- | --- |
| STAGE I vs STAGE II | ↑ | GO INTRINSIC COMPONENT OF PLASMA MEMBRANE  BENPORATH ES WITH H3K27ME3  BENPORATH EED TARGETS  BENPORATH SUZ12 TARGETS  GO ION TRANSPORT  MIKKELSEN MCV6 HCP WITH H3K27ME3  MEISSNER BRAIN HCP WITH H3K4ME3 AND H3K27ME3  MIKKELSEN MEF HCP WITH H3K27ME3  GO TRANSMEMBRANE TRANSPORTER ACTIVITY  GO TRANSPORTER ACTIVITY  CAGGTG E12 Q6  GO TRANSMEMBRANE TRANSPORT  GO BIOLOGICAL ADHESION  GO TISSUE DEVELOPMENT  GO ION TRANSMEMBRANE TRANSPORT  ZWANG TRANSIENTLY UP BY 2ND EGF PULSE ONLY  GO NEURON PART  BENPORATH PRC2 TARGETS  GO CELL CELL SIGNALING  NABA MATRISOME | 238  187  171  168  182  103  159  117  149  168  241  151  146  179  128  191  160  111  120  140 | 2.95E-75  1.36E-68  6.23E-60  2.93E-59  1.02E-56  3.37E-51  5.44E-51  6.43E-50  6.28E-48  9.56E-47  3.10E-44  4.14E-44  4.36E-44  2.70E-43  6.22E-43  1.87E-42  2.66E-42  8.82E-41  2.11E-40  2.14E-40 |
|  | ↓ | GO EXTRACELLULAR SPACE  GO DEFENSE RESPONSE  GO RESPONSE TO EXTERNAL STIMULUS  GO IMMUNE SYSTEM PROCESS  GO REGULATION OF IMMUNE SYSTEM PROCESS  GO IMMUNE RESPONSE  MODULE 137  MODULE 66  MODULE 100  GO REGULATION OF MULTICELLULAR ORGANISMAL DEVELOPMENT  GO POSITIVE REGULATION OF MULTICELLULAR ORGANISMAL PROCESS  MODULE 117  BENPORATH EED TARGETS  GO REGULATION OF CELL DIFFERENTIATION  NABA MATRISOME  MODULE 11  GO POSITIVE REGULATION OF DEVELOPMENTAL PROCESS  MODULE 88  BENPORATH ES WITH H3K27ME3  GO INNATE IMMUNE RESPONSE | 140  130  155  159  128  112  79  79  78  133  121  87  103  122  99  72  102  86  99  74 | 1.72E-52  1.69E-50  6.35E-49  5.22E-47  5.60E-43  8.92E-42  1.41E-39  2.81E-39  7.40E-39  1.56E-38  1.68E-38  2.96E-37  1.41E-36  2.30E-36  7.69E-35  8.74E-34  3.33E-33  4.79E-32  6.99E-32  1.17E-31 |
| Stage II vs Stage III | ↑ | DODD NASOPHARYNGEAL CARCINOMA UP  SENGUPTA NASOPHARYNGEAL CARCINOMA DN  ONDER CDH1 TARGETS 2 DN  GO EXTRACELLULAR SPACE  CAGGTG E12 Q6  MODULE 88  MODULE 55  GO RESPONSE TO EXTERNAL STIMULUS  GO REGULATION OF CELL PROLIFERATION  MEISSNER BRAIN HCP WITH H3K4ME3 AND H3K27ME3  CREIGHTON ENDOCRINE THERAPY RESISTANCE 2  GO RECEPTOR BINDING  GO TISSUE DEVELOPMENT  NABA MATRISOME  GO EPITHELIUM DEVELOPMENT  KOINUMA TARGETS OF SMAD2 OR SMAD3  MODULE 6  GO REGULATION OF MULTICELLULAR ORGANISMAL DEVELOPMENT  GO NEGATIVE REGULATION OF MULTICELLULAR ORGANISMAL PROCESS  GO NEGATIVE REGULATION OF RESPONSE TO STIMULUS | 161  69  67  98  125  72  70  102  92  77  53  88  88  71  68  63  46  90  68  80 | 2.87E-73  4.84E-52  2.52E-41  1.57E-34  5.48E-30  1.03E-29  3.04E-28  1.27E-27  1.27E-27  4.48E-27  5.25E-27  1.92E-25  1.30E-24  8.43E-24  9.57E-24  2.82E-23  4.28E-23  4.35E-23  7.29E-23  1.03E-22 |
|  | ↓ | BENPORATH ES WITH H3K27ME3  GO ION TRANSPORT  GO INTRINSIC COMPONENT OF PLASMA MEMBRANE  BENPORATH SUZ12 TARGETS  GO NEURON PART  GO TRANSPORTER ACTIVITY  BENPORATH EED TARGETS  GO TRANSMEMBRANE TRANSPORTER ACTIVITY  GO TRANSMEMBRANE TRANSPORT  CAGGTG E12 Q6  GO ION TRANSMEMBRANE TRANSPORT  MIKKELSEN MEF HCP WITH H3K27ME3  GO REGULATION OF TRANSPORT  GO CELL PROJECTION  GO SYNAPSE  MIKKELSEN MCV6 HCP WITH H3K27ME3  GO CELL CELL SIGNALING  GO PASSIVE TRANSMEMBRANE TRANSPORTER ACTIVITY  GO NEUROGENESIS  GO SYNAPSE PART | 248  239  274  216  230  229  208  199  208  322  175  147  258  251  158  121  157  122  213  138 | 1.50E-102  1.30E-83  2.19E-83  2.19E-83  5.57E-77  1.49E-75  2.68E-75  2.18E-73  1.11E-72  2.70E-71  1.05E-68  7.48E-67  1.14E-64  2.87E-61  8.26E-61  1.82E-60  5.59E-59  5.18E-58  2.93E-57  3.49E-57 |
| Stage I vs Stage III | ↑ | DODD NASOPHARYNGEAL CARCINOMA UP  ONDER CDH1 TARGETS 2 DN  CHARAFE BREAST CANCER LUMINAL VS BASAL DN  KOINUMA TARGETS OF SMAD2 OR SMAD3  GO TISSUE DEVELOPMENT  CAGGTG E12 Q6  SENGUPTA NASOPHARYNGEAL CARCINOMA DN  JAEGER METASTASIS DN  MEISSNER BRAIN HCP WITH H3K4ME3 AND H3K27ME3  DELYS THYROID CANCER UP  BILD HRAS ONCOGENIC SIGNATURE  GO EPITHELIUM DEVELOPMENT  ZWANG CLASS 3 TRANSIENTLY INDUCED BY EGF  MODULE 297  MODULE 357  GO INTRINSIC COMPONENT OF PLASMA MEMBRANE  WU CELL MIGRATION  NABA MATRISOME  SENESE HDAC1 TARGETS UP  MODULE 6 | 127  69  60  70  92  117  46  41  71  48  39  64  34  24  24  81  31  62  43  41 | 6.01E-55  2.85E-49  2.08E-39  6.42E-34  6.93E-34  9.26E-34  7.31E-30  9.46E-30  5.30E-28  1.86E-27  2.85E-27  1.49E-25  5.95E-24  6.32E-24  6.32E-24  1.05E-23  4.29E-23  3.73E-22  3.75E-22  7.90E-22 |
|  | ↓ | BENPORATH ES WITH H3K27ME3  MIKKELSEN MEF HCP WITH H3K27ME3  BENPORATH EED TARGETS  BENPORATH SUZ12 TARGETS  GO NEURON PART  YOSHIMURA MAPK8 TARGETS UP  ZWANG TRANSIENTLY UP BY 2ND EGF PULSE ONLY  GO NEUROGENESIS  GO SYNAPSE  MODULE 66  MODULE 100  CAGGTG E12 Q6  MODULE 137  GO CELL CELL SIGNALING  BENPORATH PRC2 TARGETS  GO SYNAPSE PART  GO REGULATION OF TRANSPORT  MODULE 11  GO SYNAPTIC SIGNALING  GO ION TRANSPORT | 160  110  140  138  146  147  168  151  109  94  92  193  91  106  97  94  158  87  78  128 | 1.53E-72  1.15E-60  1.33E-58  2.77E-58  5.32E-54  4.01E-53  2.01E-52  2.85E-52  3.60E-49  2.33E-48  4.80E-47  5.08E-46  5.08E-46  5.28E-46  9.24E-45  1.14E-44  2.12E-43  8.49E-43  1.67E-42  3.16E-41 |
