# Supplementary material for: Comparative transcriptome analysis between patient and endometrial cancer cell lines to determine common signaling pathways and markers linked to cancer progression
Source: Oncotarget. 2021 Dec 21;12(26):2500–13. doi: 10.18632/oncotarget.28161 (PMC8711572; doi:10.18632/oncotarget.28161)
Supplement: Supplementary file 3 [file oncotarget-12-2500-s003.docx]

**Supplementary Table 5: Differentially expressed genes in the top 5 signaling pathways between cancer stages.**

| Stage Comparison | Pathway | Differentially Expressed Genes (DEGs) | p-value |
| --- | --- | --- | --- |
| Stage I vs Stage II | LXR/RXR Activation | ABCG8, CYP51A1, TNFRSF11B, AMBP, KNG1, ABCG4, IL37, IL18, MLXIPL, NOS2, LDLR, IL6, A1BG, ITIH4, ABCG5, ARG2, ECHS1, TF, PLTP, TLR3, APOE, CD36, ACACA, LPL, MSR1, IL36B, MMP9, LYZ, ORM2, TTR, LBP, APOA1, FGA, ORM1, PON1, IL1RL1, AGT | 2.69E-07 |
|  | Neuroprotective Role of THOP1 in Alzheimer's Disease | DPP4, PRSS12, CMA1, PRSS50, PRSS3, KNG1, PRSS36, TMPRSS9, MME, ENDOU, CTRL, KLK1, PRSS53, PRKAR2B, TMPRSS2, GNRH1, C1R, HLA-C, HLA-B, SERPINA3, PRSS41, KLK8, TAC1, MMP9, HPN, KLK10, PRSS1, KLK11, AGT, KLK12 | 6.05E-05 |
|  | Glutamate Receptor Signaling | SLC17A7, GRIN3B, GRID2, GLS, GRIN2A, GRIN2C, GRIA3, SLC1A7, SLC1A1, GRID1, GNG7, GRIK5, GLUL, GRINA, SLC1A2, GRIK2, HOMER2, SLC17A8 | 1.50E-04 |
|  | Axonal Guidance Signaling | WNT3A, WNT10B, EFNA2, NOTUM, BMP8A, SLIT1, WNT7B, WNT6, EPHA6, BMP6, ADAMTS1, WNT1, EPHA10, BMP4, PAK3, GNAL, ADAM23, GNAT1, IRS2, NTF4, ROBO3, SDC2, NTN4, EPHA8, NTN5, TUBB1, ADAM22, UNC5A, MME, ABLIM3, BMP8B, TUBA8, WNT2B, NTNG2, WNT7A, EGF, WNT10A, NGEF, ITGA2, EFNA5, WNT8B, PRKAR2B, EPHA2, NFAT5, PRKCG, BAIAP2, GLI3, MET, GLI2, GNG13, ADAM11, ATM, FZD9, GNAZ, PIK3R2, SEMA4B, RTN4R, SEMA3B, GNG7, ADAM9, C9orf3, NFATC2, ARPC4, NTN3, FGFR2, SEMA6A, PIK3R1, CXCR4, NRP1, TUBA3C/TUBA3D, KL, TUBB, ADAMTS4, PROK1, EPHB3, GNG10, EPHA7, OPN1SW, EPHB1, MMP9, BMP2, MMP3, NTRK2, ADAMTS8 | 2.09E-04 |
|  | α-tocopherol Degradation | CYP4F3, CYP4F12, CYP4F2,  CYP4A11 | 2.77E-04 |
| Stage II vs Stage III | Calcium Signaling | SLC8A3, RCAN3, GRINA, CREB3L4, CHRNA10, NFATC2, PRKACB, ITPR1, MCU, CHRNA5, ATP2A1, CAMK2D, TPM1, CACNB2, CACNA1D, CACNB4, CACNG7, MYH3, AKAP5, CASQ1, TRPV6, TNNC1, SLC8A1, CACNG8, GRIA3, HTR3A, CAMK1, CACNA1F, SLC8A2, CHRNE, CAMK2A, CAMK2B, GRIA4, CACNA1G, CREB3L3, RYR2, GRIA1, GRIN2A, CHRNA3, TRPC6, TRPC3, CHRNB4, MYL4, TRPC1, GRIN2C, CACNG3, CHRFAM7A, RYR1, CACNA1I, MYH7B, CACNA1B, GRIN2B, CACNG5, CACNA1S | 2.94E-07 |
|  | nNOS Signaling in Skeletal Muscle Cells | SNTB1, CACNB2, SNTA1, CACNA1D, CACNB4, CAPN3, CACNG7, CACNG8, CACNA1F, CACNA1G, RYR2, CACNG3, RYR1, CACNA1I, NOS1, CACNA1B, CACNG5, CACNA1S | 1.44E-06 |
|  | Opioid Signaling Pathway | PNOC, PIK3CG, PDE1C, POMC, NOS3, PRKCQ, GRINA, MAP2K3, RGS10, AP2A2, NFKBIA, CREB3L4, MAP2K6, PRKACB, ITPR1, ADCY3, CAMK2D, CACNB2, RRAS2, CACNA1D, GNB3, CACNB4, GNAO1, ARRB1, ADCY9, CACNG7, EGR4, RGS9, GNAL, CACNG8, ADCY1, KCNJ9, SLC12A5, PRKCG, CAMK1, CACNA1F, BLK, CAMK2A, CLTCL1, CAMK2B, CACNA1G, CREB3L3, RYR2, GRIN2A, GNG7, GRIN2C, PIK3C2G, NPBWR1, CACNG3, RGS11, RYR1, CACNA1I, NOS1, CACNA1B, KCNJ3, SCN7A, GRIN2B, CACNG5, CACNA1S | 2.33E-06 |
|  | CREB Signaling in Neurons | PIK3CG, OPN1SW, PRKCQ, GNG10, CREB3L4, PRKACB, ITPR1, ADCY3, CAMK2D, FGFR3, GNG3, CACNB2, RRAS2, CACNA1D, GRM4, GNB3, CACNB4, PIK3R2, GNAO1, PIK3R5, ADCY9, CACNG7, GNAZ, GNAL, CACNG8, ADCY1, GRIA3, GRID1, PRKCG, PLCL2, GRM6, CACNA1F, CAMK2A, CAMK2B, GRIA4, CACNA1G, CREB3L3, GRIA1, GRIN2A, GNG7, GNAT1, GNG13, GRIN2C, PIK3C2G, CACNG3, GRID2, CACNA1I, CACNA1B, NOTUM, GRIN2B, CACNG5, CACNA1S | 1.06E-05 |
|  | Glutamate Receptor Signaling | GRINA, HOMER2, GLUL, SLC1A4, GRM4, GNB3, GLS, SLC1A3, GRIA3, GRID1, GRM6, SLC1A7, GRIA4, SLC17A7, GRIA1, GRIN2A, GNG7, GRIN2C, GRID2, GRIN2B | 2.65E-05 |
| Stage I vs Stage III | nNOS Signaling in Skeletal Muscle Cells | CACNA1C, SNTB1, CACNA2D2, SNTA1, RYR3, CACNA1G, RYR1, CHRNA1, CACNA1B, RYR2, CACNA1I, CACNA1A, NOS1, CACNG5 | 3.03E-06 |
|  | LXR/RXR Activation | IL1A, IL1B, PTGS2, AMBP, CYP51A1, IL18, HPX, VTN, CD36, ABCG4, ITIH4, LY96, IL36B, APOE, NGFR, CLU, ORM2, LPL, APOC1, KNG1, APOA1, ORM1, FGA, RBP4, AGT, PON1 | 9.83E-06 |
|  | FXR/RXR Activation | SLCO1B3, IL1A, IL1B, AMBP, IL18, ABCC2, SDC1, HPX, VTN, BAAT, ITIH4, IL36B, APOE, CLU, ORM2, LPL, APOC1, NR5A2, PPARG, KNG1, APOA1, ORM1, FGA, RBP4, AGT, PON1 | 1.81E-05 |
|  | Neuroprotective Role of THOP1 in Alzheimer's Disease | DPP4, PNOC, PRSS12, PRKAR2B, PRSS27, HGFAC, GZMM, HLA-B, GNRH2, HPN, HLA-F, PRSS50, PRSS41, KLK11, PRSS1, KNG1, MAPT, KLK15, KLK1, KLK12, KLK8, PRSS57, AGT | 6.64E-05 |
|  | Calcium Signaling | SLC8A3, CACNA1C, ASPH, ITPR3, CACNA2D2, GRIN3B, PRKAR2B, CREB5, CHRNE, PNCK, CAMK1, RYR3, CACNA1G, RYR1, GRIN2C, CAMK2B, TNNI2, CHRNA1, CACNA1B, CHRNG, RYR2, CACNA1I, CASQ1, MYL4, HTR3A, CREB3L3, CACNA1A, CHRNB2, GRIA4, TRPC7, GRIA1, TRDN, CACNG5, GRIN2B | 7.26E-05 |
