# Supplementary material for: Comparative transcriptome analysis between patient and endometrial cancer cell lines to determine common signaling pathways and markers linked to cancer progression
Source: Oncotarget. 2021 Dec 21;12(26):2500–13. doi: 10.18632/oncotarget.28161 (PMC8711572; doi:10.18632/oncotarget.28161)
Supplement: Supplementary file 4 [file oncotarget-12-2500-s004.docx]

**Supplementary Table 6: Notable biological processes involved in each zone of Venn diagram; significant (FDR <0.05).**

| Zone | Gene Set Name | # of Genes | FDR  q-value |
| --- | --- | --- | --- |
| 1164 | GO IMMUNE SYSTEM PROCESS | 120 | 3.7E-27 |
|  | GO INTRINSIC COMPONENT OF PLASMA MEMBRANE | 108 | 3.7E-27 |
|  | GO RESPONSE TO EXTERNAL STIMULUS | 106 | 1.06E-22 |
|  | BENPORATH EED TARGETS | 79 | 1.06E-22 |
|  | GO REGULATION OF IMMUNE SYSTEM PROCESS | 90 | 8.95E-22 |
|  | GO DEFENSE RESPONSE | 83 | 2.47E-21 |
|  | BENPORATH SUZ12 TARGETS | 75 | 7.04E-21 |
|  | GO BIOLOGICAL ADHESION | 74 | 2E-20 |
|  | GO REGULATION OF MULTICELLULAR ORGANISMAL DEVELOPMENT | 96 | 3.57E-20 |
|  | NABA MATRISOME | 72 | 2.59E-19 |
|  | GO REGULATION OF TRANSPORT | 97 | 1.78E-18 |
|  | GO EXTRACELLULAR SPACE | 82 | 5.93E-18 |
|  | GO POSITIVE REGULATION OF MULTICELLULAR ORGANISMAL PROCESS | 82 | 1.27E-17 |
|  | MEISSNER BRAIN HCP WITH H3K4ME3 AND H3K27ME3 | 70 | 3.03E-17 |
|  | GO HOMEOSTATIC PROCESS | 78 | 1.61E-16 |
|  | GO POSITIVE REGULATION OF IMMUNE SYSTEM PROCESS | 61 | 2.57E-16 |
|  | BENPORATH ES WITH H3K27ME3 | 70 | 2.81E-16 |
|  | GO SMALL MOLECULE METABOLIC PROCESS | 91 | 3.63E-16 |
|  | GO TISSUE DEVELOPMENT | 83 | 4.14E-16 |
|  | ZWANG TRANSIENTLY UP BY 2ND EGF PULSE ONLY | 89 | 7.43E-16 |
| 933 | CAGGTG E12 Q6 | 127 | 4.91E-28 |
|  | DODD NASOPHARYNGEAL CARCINOMA UP | 104 | 2.96E-26 |
|  | GO ION TRANSPORT | 78 | 4.66E-21 |
|  | BENPORATH ES WITH H3K27ME3 | 69 | 2.31E-18 |
|  | GO CELL PROJECTION | 87 | 3.60E-17 |
|  | GO PLASMA MEMBRANE REGION | 59 | 4.54E-16 |
|  | GO CELL DEVELOPMENT | 74 | 7.98E-16 |
|  | GO MEMBRANE REGION | 65 | 8.59E-16 |
|  | GO TRANSPORTER ACTIVITY | 69 | 1.27E-15 |
|  | GO REGULATION OF TRANSPORT | 84 | 1.40E-15 |
|  | CAGCTG AP4 Q5 | 76 | 1.53E-15 |
|  | GO ENDOPLASMIC RETICULUM | 78 | 5.13E-15 |
|  | AACTTT UNKNOWN | 85 | 5.33E-15 |
|  | GO TRANSMEMBRANE TRANSPORTER ACTIVITY | 59 | 5.35E-15 |
|  | SENGUPTA NASOPHARYNGEAL CARCINOMA DN | 35 | 5.80E-15 |
|  | GO TRANSMEMBRANE TRANSPORT | 62 | 6.10E-15 |
|  | GO INTRINSIC COMPONENT OF PLASMA MEMBRANE | 78 | 6.43E-15 |
|  | BENPORATH SUZ12 TARGETS | 60 | 6.43E-15 |
|  | GO CATION TRANSPORT | 51 | 3.17E-14 |
|  | GGGAGGRR MAZ Q6 | 93 | 3.41E-14 |
| 499 | ZWANG TRANSIENTLY UP BY 2ND EGF PULSE ONLY | 59 | 3.07E-15 |
|  | GGGAGGRR MAZ Q6 | 65 | 1.48E-13 |
|  | CAGGTG E12 Q6 | 68 | 1.48E-13 |
|  | YOSHIMURA MAPK8 TARGETS UP | 46 | 3.55E-12 |
|  | BENPORATH EED TARGETS | 41 | 4.91E-12 |
|  | GO TRANSPORTER ACTIVITY | 45 | 4.91E-12 |
|  | GO INTRINSIC COMPONENT OF PLASMA MEMBRANE | 51 | 8.96E-12 |
|  | GO CELL PROJECTION | 53 | 1.14E-11 |
|  | BENPORATH ES WITH H3K27ME3 | 41 | 1.77E-11 |
|  | GO CELL JUNCTION | 41 | 4.17E-11 |
|  | DODD NASOPHARYNGEAL CARCINOMA UP | 52 | 6.94E-11 |
|  | GO NEUROGENESIS | 45 | 6.96E-11 |
|  | BENPORATH SUZ12 TARGETS | 38 | 1.31E-10 |
|  | GO EXTRACELLULAR SPACE | 44 | 1.33E-10 |
|  | GO TRANSMEMBRANE TRANSPORTER ACTIVITY | 37 | 1.63E-10 |
|  | CTTTGT LEF1 Q2 | 53 | 2.85E-10 |
|  | NABA MATRISOME | 37 | 3.58E-10 |
|  | GO ION TRANSPORT | 41 | 4.58E-10 |
|  | GO TRANSMEMBRANE TRANSPORT | 38 | 4.96E-10 |
|  | GO CALCIUM ION BINDING | 30 | 6.98E-10 |
| 241 | BENPORATH ES WITH H3K27ME3 | 39 | 4.02E-19 |
|  | MIKKELSEN MEF HCP WITH H3K27ME3 | 29 | 1.38E-17 |
|  | BENPORATH EED TARGETS | 31 | 9.43E-13 |
|  | MIKKELSEN MCV6 HCP WITH H3K27ME3 | 21 | 4.65E-12 |
|  | NABA MATRISOME | 29 | 1.40E-11 |
|  | ZWANG TRANSIENTLY UP BY 2ND EGF PULSE ONLY | 36 | 4.24E-11 |
|  | GGGAGGRR MAZ Q6 | 41 | 5.44E-11 |
|  | BENPORATH SUZ12 TARGETS | 28 | 8.14E-11 |
|  | CAGGTG E12 Q6 | 42 | 1.68E-10 |
|  | GO NEURON PART | 29 | 1.26E-09 |
|  | GO REGULATION OF MEMBRANE POTENTIAL | 16 | 8.14E-09 |
|  | MEISSNER NPC HCP WITH H3K4ME2 AND H3K27ME3 | 16 | 9.68E-09 |
|  | GO NEUROGENESIS | 29 | 1.10E-08 |
|  | YOSHIMURA MAPK8 TARGETS UP | 28 | 1.10E-08 |
|  | MEISSNER BRAIN HCP WITH H3K4ME3 AND H3K27ME3 | 25 | 2.35E-08 |
|  | BENPORATH PRC2 TARGETS | 20 | 2.35E-08 |
|  | GO SYSTEM PROCESS | 32 | 2.73E-08 |
|  | GCANCTGNY MYOD Q6 | 23 | 3.99E-08 |
|  | MEISSNER NPC HCP WITH H3 UNMETHYLATED | 18 | 4.72E-08 |
|  | GO CELL CELL SIGNALING | 21 | 4.72E-08 |
| 1410 | BENPORATH ES WITH H3K27ME3 | 128 | 4.56E-46 |
|  | BENPORATH EED TARGETS | 123 | 6.31E-45 |
|  | GO INTRINSIC COMPONENT OF PLASMA MEMBRANE | 145 | 2.39E-39 |
|  | BENPORATH SUZ12 TARGETS | 114 | 2.39E-39 |
|  | GO EXTRACELLULAR SPACE | 118 | 1.95E-30 |
|  | GO ION TRANSPORT | 111 | 1.75E-29 |
|  | GO BIOLOGICAL ADHESION | 97 | 1.04E-27 |
|  | MEISSNER BRAIN HCP WITH H3K4ME3 AND H3K27ME3 | 98 | 2.93E-27 |
|  | BENPORATH PRC2 TARGETS | 76 | 2.93E-27 |
|  | MIKKELSEN MCV6 HCP WITH H3K27ME3 | 62 | 9.84E-27 |
|  | NABA MATRISOME | 95 | 9.84E-27 |
|  | GO REGULATION OF TRANSPORT | 130 | 9.84E-27 |
|  | CAGGTG E12 Q6 | 157 | 1.35E-26 |
|  | GO TRANSMEMBRANE TRANSPORTER ACTIVITY | 92 | 7.67E-26 |
|  | GO CELL PROJECTION | 127 | 1.34E-25 |
|  | AACTTT UNKNOWN | 131 | 1.82E-25 |
|  | GO TRANSPORTER ACTIVITY | 104 | 3.59E-25 |
|  | GO REGULATION OF MULTICELLULAR ORGANISMAL DEVELOPMENT | 121 | 5.19E-25 |
|  | GO RESPONSE TO EXTERNAL STIMULUS | 127 | 6.40E-25 |
|  | GO NEURON PART | 102 | 2.42E-24 |
| 561 | MODULE 137 | 47 | 6.09E-27 |
|  | MODULE 66 | 47 | 6.09E-27 |
|  | MODULE 100 | 46 | 2.41E-26 |
|  | YOSHIMURA MAPK8 TARGETS UP | 58 | 1.30E-19 |
|  | CAGGTG E12 Q6 | 79 | 8.53E-19 |
|  | MODULE 11 | 38 | 9.91E-19 |
|  | MIKKELSEN MEF HCP WITH H3K27ME3 | 39 | 2.19E-18 |
|  | GO EXTRACELLULAR SPACE | 56 | 2.42E-17 |
|  | GO CELL CELL SIGNALING | 42 | 5.03E-17 |
|  | ZWANG TRANSIENTLY UP BY 2ND EGF PULSE ONLY | 62 | 6.78E-17 |
|  | GO DEFENSE RESPONSE | 52 | 9.62E-17 |
|  | GO INTRINSIC COMPONENT OF PLASMA MEMBRANE | 60 | 1.36E-16 |
|  | BENPORATH ES WITH H3K27ME3 | 48 | 1.28E-15 |
|  | GO ION TRANSPORT | 50 | 5.79E-15 |
|  | GO REGULATION OF TRANSPORT | 60 | 7.59E-15 |
|  | GO RESPONSE TO EXTERNAL STIMULUS | 60 | 1.10E-14 |
|  | GO NEURON DIFFERENTIATION | 41 | 1.84E-14 |
|  | GO SYNAPSE | 38 | 2.70E-14 |
|  | GO CELLULAR RESPONSE TO ORGANIC SUBSTANCE | 59 | 7.49E-14 |
|  | ONDER CDH1 TARGETS 2 DN | 30 | 8.25E-14 |
| 757 | BENPORATH ES WITH H3K27ME3 | 83 | 2.05E-32 |
|  | CAGGTG E12 Q6 | 121 | 2.74E-31 |
|  | MIKKELSEN MEF HCP WITH H3K27ME3 | 61 | 3.13E-31 |
|  | BENPORATH SUZ12 TARGETS | 78 | 3.60E-31 |
|  | MIKKELSEN MCV6 HCP WITH H3K27ME3 | 52 | 1.64E-29 |
|  | GO NEURON PART | 83 | 2.46E-29 |
|  | MEISSNER BRAIN HCP WITH H3K4ME3 AND H3K27ME3 | 75 | 3.95E-28 |
|  | GO SYNAPSE | 62 | 1.44E-26 |
|  | YOSHIMURA MAPK8 TARGETS UP | 80 | 2.46E-26 |
|  | BENPORATH EED TARGETS | 72 | 4.09E-26 |
|  | GO NEUROGENESIS | 82 | 9.08E-26 |
|  | GO CELL PROJECTION | 91 | 1.44E-24 |
|  | GO SYNAPSE PART | 52 | 8.10E-23 |
|  | GO CELL CELL SIGNALING | 57 | 2.93E-22 |
|  | GO TISSUE DEVELOPMENT | 80 | 2.93E-22 |
|  | GO INTRINSIC COMPONENT OF PLASMA MEMBRANE | 83 | 6.39E-22 |
|  | ZWANG TRANSIENTLY UP BY 2ND EGF PULSE ONLY | 85 | 6.39E-22 |
|  | GO REGULATION OF TRANSPORT | 87 | 6.79E-22 |
|  | GGGAGGRR MAZ Q6 | 98 | 2.03E-21 |
|  | AACTTT UNKNOWN | 87 | 1.39E-20 |
